# A diet-induced animal model of non-alcoholic fatty liver disease and hepatocellular cancer

**DOI:** 10.1016/j.jhep.2016.05.005

**Published:** 2016-05-31

**Authors:** Amon Asgharpour, Sophie C. Cazanave, Tommy Pacana, Mulugeta Seneshaw, Robert Vincent, Bubu A. Banini, Divya Prasanna Kumar, Kalyani Daita, Hae-Ki Min, Faridoddin Mirshahi, Pierre Bedossa, Xiaochen Sun, Yujin Hoshida, Srinivas V. Koduru, Daniel Contaifer, Urszula Osinska Warncke, Dayanjan S. Wijesinghe, Arun J. Sanyal

**Affiliations:** 1 Division of Gastroenterology, Hepatology and Nutrition, Department of Internal Medicine, Virginia Commonwealth University, Richmond, VA 23298-0341, USA; 2 Department of Pathology, Hospital Beaujon, University Paris-Diderot, Paris, France; 3 Division of Liver Diseases, Department of Medicine, Liver Cancer Program, Tisch Cancer Institute, Icahn School of Medicine at Mount Sinai, New York, NY 10029, USA; 4 Gene Arrays, Entity of Vedic Research, Inc, New York, NY 11783, USA; 5 Department of Pharmacotherapy and Outcomes Science, School of Pharmacy, Virginia Commonwealth University, Richmond, VA 23298, USA; 6 Department of Surgery, Virginia Commonwealth University, Richmond, VA 23298, USA

**Keywords:** Steatosis, Fibrosis, Hepatocyte ballooning, Hepatocellular carcinoma, Drug therapy

## Abstract

**Background & Aims:**

The lack of a preclinical model of progressive non-alcoholic steatohepatitis (NASH) that recapitulates human disease is a barrier to therapeutic development.

**Methods:**

A stable isogenic cross between C57BL/6J (B6) and 129S1/SvImJ (S129) mice were fed a high fat diet with *ad libitum* consumption of glucose and fructose in physiologically relevant concentrations and compared to mice fed a chow diet and also to both parent strains.

**Results:**

Following initiation of the obesogenic diet, B6/129 mice developed obesity, insulin resistance, hypertriglyceridemia and increased LDL-cholesterol. They sequentially also developed steatosis (4–8 weeks), steatohepatitis (16–24 weeks), progressive fibrosis (16 weeks onwards) and spontaneous hepatocellular cancer (HCC). There was a strong concordance between the pattern of pathway activation at a transcriptomic level between humans and mice with similar histological phenotypes (FDR 0.02 for early and 0.08 for late time points). Lipogenic, inflammatory and apoptotic signaling pathways activated in human NASH were also activated in these mice. The HCC gene signature resembled the S1 and S2 human subclasses of HCC (FDR 0.01 for both). Only the B6/129 mouse but not the parent strains recapitulated all of these aspects of human NAFLD.

**Conclusions:**

We here describe a diet-induced animal model of non-alcoholic fatty liver disease (DIAMOND) that recapitulates the key physiological, metabolic, histologic, transcriptomic and cell-signaling changes seen in humans with progressive NASH.

**Lay summary:**

We have developed a diet-induced mouse model of non-alcoholic steatohepatitis (NASH) and hepatic cancers in a cross between two mouse strains (129S1/SvImJ and C57Bl/6J). This model mimics all the physiological, metabolic, histological, transcriptomic gene signature and clinical endpoints of human NASH and can facilitate preclinical development of therapeutic targets for NASH.

## Introduction

Non-alcoholic fatty liver disease (NAFLD) has emerged as the leading cause of chronic liver disease in most parts of the Western world. NAFLD includes both a fatty liver and steatohepatitis which can progress through stages of fibrosis to cirrhosis and can be complicated by hepatocellular cancer [1]. There is currently no approved therapy for NASH.

There is a need to create animal models that recapitulate the physiology, histology, outcomes and transcriptomic changes seen in humans with NASH. While a large number of models have been described, they have several limitations [2] (Supplementary Table 1). These models are dissimilar to human NASH by either requiring specific gene knockout, non-physiological dietary manipulations or their lack of insulin resistance or liver histology typical of human NASH [3]. Importantly, most models do not develop progressive fibrosis and do not lead to hepatocellular cancer [2,4]. While the Ossabaw pig develops lesions very consistent with human NASH [5], the cost and resources needed to use this model renders it challenging for widespread use. The C57BL/6J mice fed a high fat, high sugar diet develop steatohepatitis with fibrosis [6]. However, the severity of fibrosis is mild and the disease is not complicated by hepatocellular cancer, an increasingly recognized and clinically important end-point of the progression of NASH.

An ideal preclinical model for NASH should be relatively simple, triggered by the same causes as human disease (caloric excess), associated with the same risk factors as in humans (obesity, insulin resistance and dyslipidemia), and it should match human disease with respect to metabolic features, histology, outcomes, gene expression signature, lipid accumulation and activation of pathways relevant in humans. Importantly, it should also recapitulate the various histological stages of human disease. The development of hepatocellular carcinoma (HCC) in this setting should also be triggered by the disease state and not by administration of a chemical carcinogen. We here report a diet-induced animal model of non-alcoholic fatty liver disease (DIAMOND) using an isogenic strain derived from a cross of two common mouse strains, 129S1/SvImJ and C57BL/6J where a simple high fat diet accompanied by *ad libitum* consumption of water with a high fructose and glucose content (Western diet sugar water (WD SW)) sequentially induces steatosis, steatohepatitis, progressive fibrosis and HCC. The phenotype was noted serendipitously and refined to develop the isogenic strain and the dietary manipulations to reproducibly produce the disease phenotype.

## Materials and methods

### Animals

A unique, isogenic mouse strain derived from a C57BL/6J and 129S1/SvImJ background (B6/129) was created and maintained with inbreeding. Additional information on B6/129 origin are described in the Supplementary materials and methods section. Pure C57BL/6J (B6) or 129S1/SvImJ (S129) were purchased from Jackson Laboratory (Bar Harbor) and used as controls. All mice were housed in a 12 h light–12 h dark cycle in a 21–23 °C facility and were euthanized at varying time points following initiation of dietary intervention. All procedures were performed according to protocols approved by the Animal Care and Use Committee of Virginia Commonwealth University (IACUC AM 10154).

### Dietary interventions

Male mice (8–12 weeks of age) were fed *ad libitum* a high fat diet, high carbohydrate diet (Western diet, WD) with 42% kcal from fat and containing 0.1% cholesterol (Harlan TD.88137) with a high fructose-glucose solution (SW, 23.1 g/L d-fructose +18.9 g/L d-glucose), as previously described [6]. Control mice were fed a standard chow diet (CD, Harlan TD.7012) with normal water (NW).

### Histological analysis

Liver histology was assessed using hematoxylin and eosin (H&E) stains in paraffin-embedded sections using standard commercially used methods. Fibrosis was assessed using both Masson’s trichrome and Sirius Red stains in paraffin-embedded sections using established methodology [7]. The presence of steatosis was further confirmed used Oil-Red-O stains in frozen sections using standard methods [8]. The liver histology was evaluated by an expert liver pathologist (PB) who was blinded to the dietary condition. Histology was assessed using the NASH CRN and fatty liver inhibition of progression (FLIP) consortia criteria (see the Supplementary materials and methods section for details) [3].

A detailed description of biochemical, molecular, transcriptomic, bioinformatics and statistical analysis is also provided in the Supplementary materials and methods section.

## Results

### The genetic background of the mouse

Following repeated brother-sister mating of the C57BL/6J;129S1/SvImJ mice (B6/129) over a four-year time frame, it was observed that the histological phenotype of steatohepatitis could be obtained consistently. A set of 158 single nucleotide polymorphisms (SNPs) that included both specific C57BL/6 and 129S1 SNPs was tested in a random set of six mice (Supplementary Table 2). These demonstrated that approximately 60% of the SNPs were of C57BL/6 origin while the rest were of 129S1 origin. Importantly, all mice were genetically identical confirming the isogenic nature of the strain. The mice have been re-derived and their genotype and phenotype were found to be maintained.

### DIAMOND mice develop obesity, liver injury, dyslipidemia and insulin resistance

**Fig. 1:**
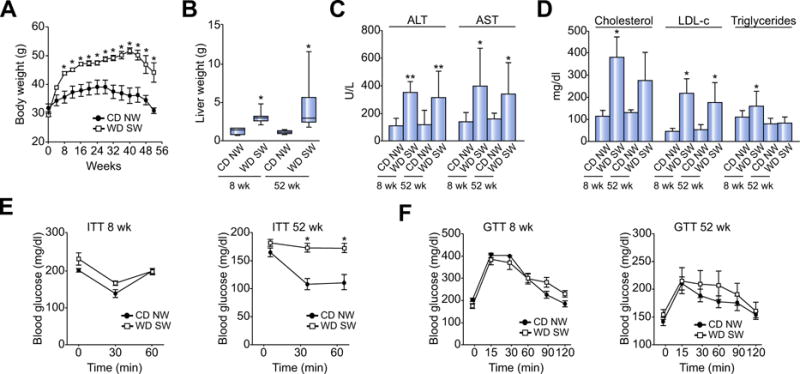
DIAMOND mice develop obesity, liver injury, dyslipidemia and insulin resistance B6/129 mice were fed a chow diet (CD NW) or high fructose/glucose, high fat Western Diet (WD SW) for up to 52 weeks. (A) Body weight change over time, (B) Liver weight, (C) serum ALT and AST levels, (D) serum cholesterol, LDL-c and triglycerides levels, (E) Insulin tolerance test (ITT) and (F) glucose tolerance test (GTT). Data are expressed as the mean ± SEM for 6–10 mice per group;*p <0.05 and **p <0.001, WD SW compared to CD NW.

B6/129 mice fed a WD SW developed rapid weight gain and obesity compared to CD NW-fed control mice (Fig. 1A). This was accompanied by an increase in liver weight at all time points (data for weeks 8 and 52 shown in Fig. 1B). Feeding a WD SW diet also led to a significant increase in AST and ALT which persisted up to 52 weeks of diet administration (Fig. 1C). Mice fed a WD SW diet also developed an increase in total cholesterol and LDL-cholesterol (LDL-c), as it has been reported in humans with NASH [9]. Hypertriglyceridemia also developed in these mice and was maximal at 8 weeks (Fig. 1D). At week 52, LDL-c remained significantly elevated while the hypertriglyceridemia declined to values noted in CD NW-fed mice.

Insulin resistance was assessed using an insulin tolerance test (ITT). Although WD SW-fed mice remained initially insulin sensitive at 8 weeks, they developed significant insulin resistance at 16 weeks which was sustained up to 52 weeks (Figs. 1E, 7B). On a separate day, a glucose tolerance test (GTT) was also performed. While glucose levels were often higher following intraperitoneal glucose administration in mice fed WD SW (Fig. 1F), these differences did not reach statistical significance. This suggested that despite insulin resistance, the mice still had enough pancreatic islet β cell reserve to be able to mount a glucoregulatory insulin response after a glucose challenge. There was also a trend towards decreased circulating adiponectin 8 weeks after WD SW initiation which remained relatively unchanged thereafter (Supplementary Fig. 1). Thus, these results indicate that B6/129 mice fed a WD SW diet develop obesity, dyslipidemia, liver injury and insulin resistance.

### DIAMOND mice sequentially develop a fatty liver, steatohepatitis and advanced fibrosis

**Fig. 2:**
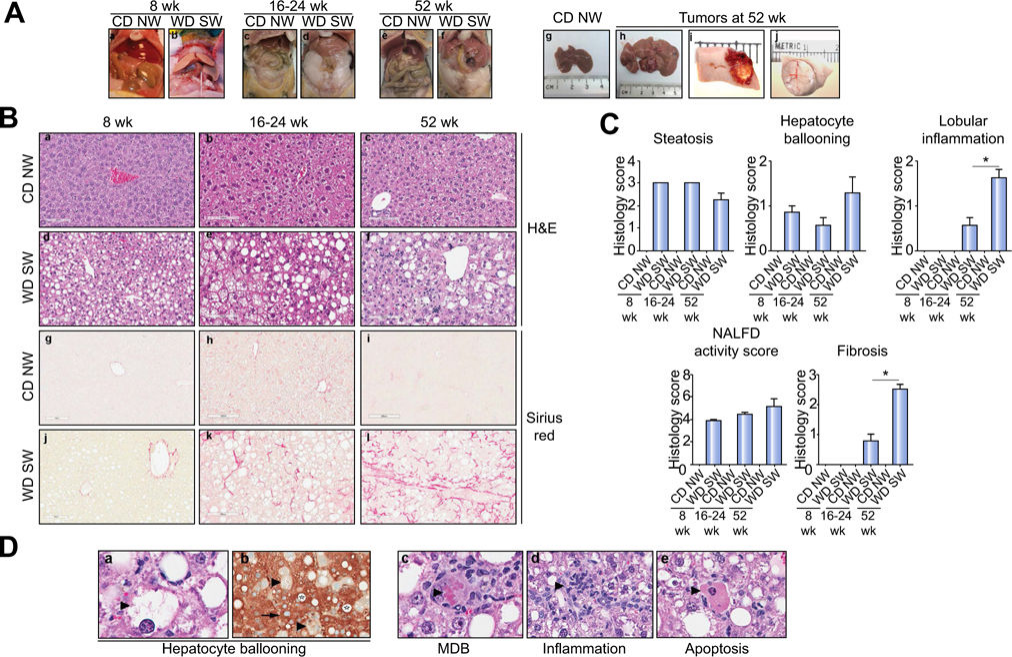
DIAMOND mice sequentially develop a fatty liver, steatohepatitis, advanced fibrosis and liver tumors (A) Gross liver from B6/129 mice fed a chow diet (CD NW) or high fructose/glucose, high fat Western Diet (WD SW) for 8 (a, b), 16–24 (c, d) and 52 weeks (e, f). In mice fed a high fat Western Diet for 52 weeks, multiple foci of tumors were observed at the time of necropsy (h–j) as compared to CD NW mice (g); areas of hemorrhage were seen in larger tumors (i). (B) Microscopic views of livers from CD NW or WD SW mice at 8 (a, d, g, j), 16–24 (b, e, h, k) or 52 weeks (c, f, i, l) of diet. Representative liver sections stained with hematoxylin-eosin (H&E) (a–f) or Picrosirius Red (g–l) are shown. Original magnification, ×20. (C) Histology score for steatosis, hepatocyte ballooning, lobular inflammation, NAFLD Activity Score and fibrosis were quantified. Data are expressed as the mean ± SEM for 6–10 mice per group; *p <0.05. (D) Representative images of hematoxylin-eosin (H&E) (a, c–e) and CK-18 (b) staining of liver tissue from mice fed a WD SW for 52 weeks depicting the individual component of steatohepatitis (as indicated by large arrow): (a) hepatocyte ballooning, (c) Mallory-Denk bodies (MDB), (d) lobular inflammation and (e) apoptotic bodies. (b) A marked CK-18 staining is present in the cytoplasm of normal hepatocytes (as indicated by small arrow), whereas in ballooned hepatocyte (as indicated by large arrow), a reduction (almost a complete loss) in CK-18 staining in their cytoplasm is noted. Lipid vacuoles are indicated by a star. Original magnification, ×40.

Within 4–8 weeks of WD SW administration, the liver became tan and visibly lighter in color compared to CD NW-fed mice and this was maintained at 16–24 and 52 weeks (Fig. 2A). At week 52, the surface of the liver demonstrated nodularity in some but not in all mice and multiple foci of tumors with hemorrhage within some of the larger tumors were seen at the time of necropsy.

Liver histology indicated that all WD SW-fed mice developed extensive macrovesicular and small droplet steatosis by week 8 (Fig. 2 and Supplementary Fig. 2). The latter were seen most commonly in zone 3. Most mice had grade 3 steatosis at 8 weeks but this declined after 32 weeks. These findings were confirmed by Oil-Red-O staining and by direct triglyceride quantification (Supplementary Fig. 2).

**Fig. 3:**
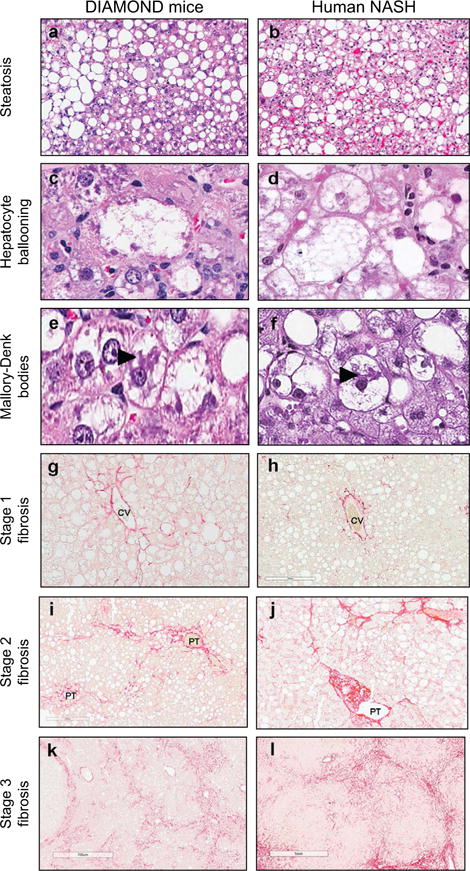
Main histological features of DIAMOND mice are comparable to human NASH Representative images of liver histology from DIAMOND mice (a,c,e,g,i,k) or human NASH (b, d, f, h, j, l) depicting steatosis (a, b; H&E; original magnification, ×5), Hepatocyte ballooning (c, d; H&E; original magnification, ×40), Mallory-Denk bodies (as indicated by large arrow) (e, f; H&E; original magnification, ×40), Stage 1 fibrosis (g, h; Sirius Red; original magnification, ×20), Stage 2 fibrosis (i, j; Sirius Red; original magnification, ×20), Stage 3 fibrosis (k, l; Sirius Red; original magnification, ×10). CV, central vein; PT, portal tract.

Steatohepatitis developed in an increasing proportion of mice after week 8 and became pronounced after 16–24 weeks following initiation of WD SW diet. This was characterized by steatosis, lobular inflammation and hepatocellular ballooning some with Mallory-Denk bodies (MDB) (Fig. 2B–D). Histologically, the ballooning, Mallory-Denk bodies and apoptotic cells were as typically described in the literature and comparable to human NASH (Figs. 2D and 3). In contrast to the decrease in steatosis, ballooning and inflammation were prominent at week 52. Cytokeratin-18 (CK-18) stains revealed normal staining in histologically normal hepatocytes and clearing within ballooned cells as described in humans (Fig. 2D) [10].

The NAFLD activity score (NAS) increased by week 8 and remained significantly higher than chow-fed mice by week 52 (Fig. 2C). At earlier time points, steatosis contributed disproportionately to the NAS while at late time points, inflammation and ballooning were the major contributors.

Pericellular stage 1 sinusoidal fibrosis, as assessed by Sirius Red staining, was present by weeks 16–24 after initiation of the WD SW diet, with also stage 2 fibrosis observed in some mice. Fibrosis increased progressively and by week 52, severe bridging fibrosis was noted in the majority of mice with early nodule formation in some (Figs. 2B, 3 and Supplementary Fig. 3A). The fibrosis pattern resembled that seen in humans with stages 1–3 patterns of fibrosis identified in the mice (Fig. 3). Collagen fibrils could be seen in the sinusoids at high magnification and the activation of fibrogenesis was further confirmed by α-smooth muscle actin and desmin stains (Supplementary Fig. 3B, C) [11]. Quantitative morphometry further demonstrated that the collagen proportional area increased to 2.3 ± 0.4% by week 52 in WD SW-fed mice. Collectively, these results indicate that B6/129 mice fed a WD SW diet sequentially developed steatosis, steatohepatitis and progressive fibrosis (16 weeks onwards).

### Activation of signaling pathways relevant to human NASH

**Fig. 4:**
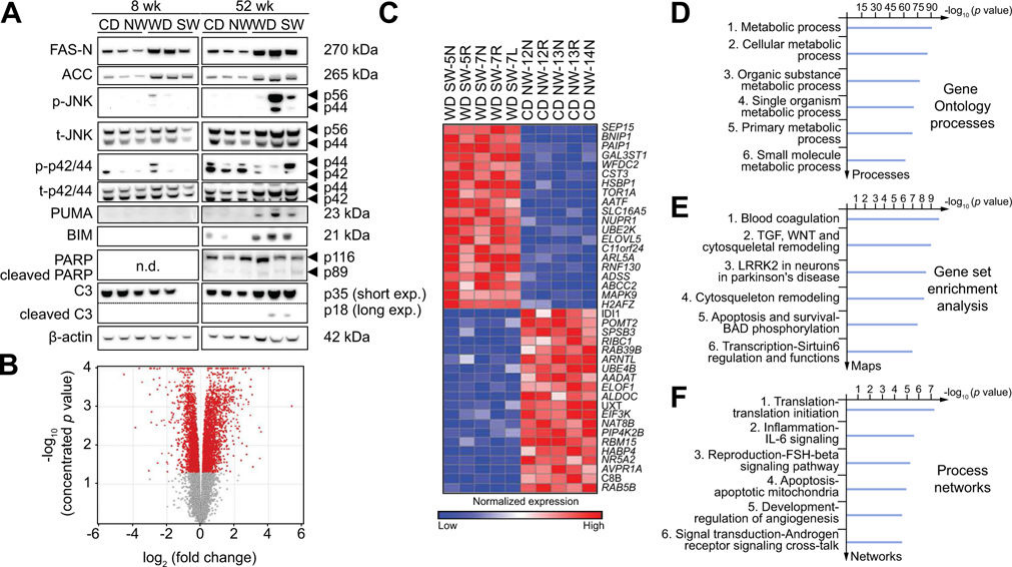
Activation of signaling pathways relevant to human NASH in the liver of DIAMOND mice (A) Whole cell lysates were prepared from liver tissue from B6/129 mice fed a chow diet (CD NW) or high fructose/glucose, high fat Western Diet (WD SW) for 8 or 52 weeks. Immunoblot analysis were performed for FAS-N, acetyl-CoA carboxylase (ACC), phosphorylated and total JNK (p-JNK and t-JNK), phosphorylated and total p42/p44 (p-p42/p44 and t-p42/p44), PUMA, BIM, PARP displaying cleaved PARP product p89 and caspase-3 (C3) displaying cleaved caspase-3 product p18. The cleaved form of C3 was only visualized after long exposure times. β-actin was used as a control for protein loading. Bands were cut and combined from the same radiograph. (B–F) Transcriptome analysis was performed on liver tissues from CD NW or WD SW mice after 8 weeks of diet (n = 5 per group). The data are presented as: (B) volcano plot; (C) Heat map demonstrating deregulated genes. Red and blue colors indicate high and low gene expression, respectively; (D) Gene ontology (GO) processes; (E) Gene set Enrichment Analysis (GSEA); and (F) Process Networks analysis. The top rank ordered processes, maps and networks are based on statistical significance.

Increased lipogenic-, inflammatory- and pro-apoptotic signaling are hallmarks of NASH in humans [12–14]. At both weeks 8 and 52, there was a significant increase in expression of fatty acid synthetase (FAS) as well as acetyl CoA carboxylase (ACC) (Fig. 4A). Endoplasmic reticulum (ER) stress was evident in the livers from WD SW-fed mice with the detection of an active phosphorylation of PERK by 8 weeks and increased *CHOP* mRNA expression at later time point (52 weeks) (Supplementary Fig. 4A, B). However, IRE-1 activity as assessed by Xbp-1 splicing remained unchanged by high fat diet throughout the time course of the unfolded protein response (UPR) (Supplementary Fig. 4C). Phosphorylated and activated JNK, a key mediator of inflammation in human NASH [12,15], significantly increased after week 8 up to week 52. Activation of apoptotic signaling relevant to human NASH and lipotoxicity [16,17] via increased expression in pro-apoptotic proteins BIM and p53 upregulated modulator of apoptosis (PUMA), and cleaved poly ADP ribose polymerase (PARP) and caspase 3 was observed at later time points (data for week 52 shown in Fig. 4A).

### DIAMOND mice have a transcriptomic profile similar to humans with NASH

Volcano plot and heat map visualization of the hepatic transcriptome demonstrated distinct differences between CD NW-fed and WD SW-fed mouse at 8 weeks (Fig. 4B, C). The separation of gene expression profile was further confirmed by principal component analysis. There was also a remarkable similarity in gene expression signatures from one mouse to the other within each experimental group. Gene Ontology™ evaluation indicated a major effect of genes involved in multiple metabolic processes by week 8 in WD SW-fed mice (Fig. 4D). Gene Set Enrichment Analysis, based on *a priori* defined sets of genes along specific pathways, demonstrated multiple pathways that were differentially activated with blood coagulation, TGF-β-Wnt, cytoskeletal remodeling and LRRK2 in Parkinsons disease as the top four pathways based on statistical significance (Fig. 4E and Supplementary Fig. 5). There was also evidence for increased apoptotic and inflammatory signaling pathway activation at a transcriptional level. Biological networks activated by WD SW was further determined (Fig. 4F) in an unbiased manner using the Analyze Networks algorithm [18,19]. A β-catenin, JAK2, SMAD3, TGF-β receptor type II, ILK network that impacts cell proliferation, regulation of phosphorylation and macromolecule metabolic processes was the principal network upregulated by WD SW at 8 weeks (Supplementary Table 3).

**Fig. 5:**
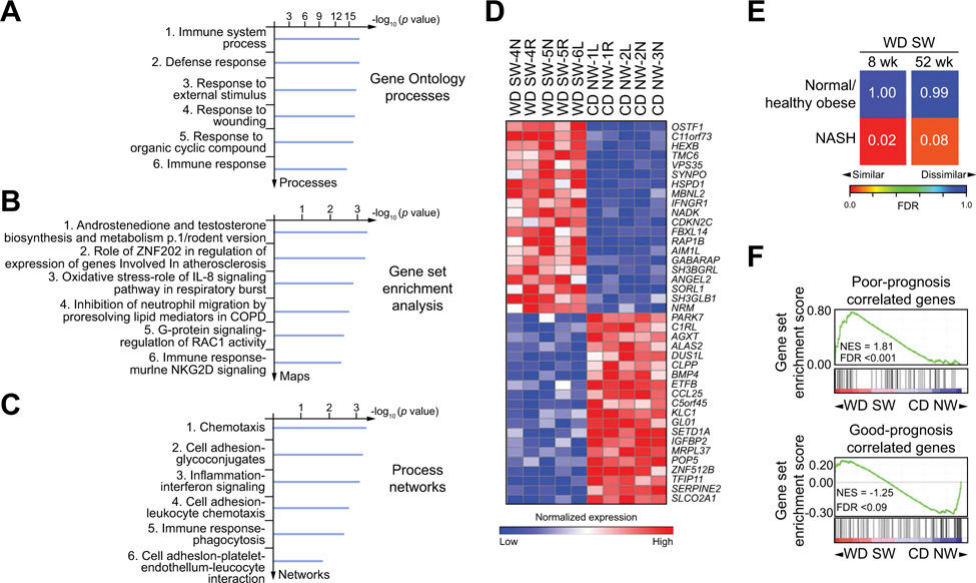
Hepatic gene expression dataset in DIAMOND mice at 52 weeks concords with a human liver cirrhosis and NASH-associated gene signature Transcriptome analysis was performed on liver tissues from B6/129 mice fed a chow diet (CD NW) or a high fructose/glucose, high fat Western diet (WD SW) mice for 52 weeks (A–F) and 8 weeks (E) (n = 5 per group). (A) Gene ontology (GO) processes; (B) Gene set Enrichment Analysis (GSEA); (C) Process Networks analysis. The top rank ordered processes, maps and networks are based on statistical significance; (D) Heat map demonstrating deregulated genes. Red and blue colors indicate high and low gene expression, respectively. (E) Similarity between global liver transcriptome of mice fed a WD SW for 8 or 52 weeks and global liver transcriptome in liver biopsy tissues from 18 human NASH patients and 41 normal/healthy obese individuals using subclass mapping algorithm (see Supplementary materials and methods). Numbers on heat map indicate FDR values for the transcriptome similarity. (F) Concordance by GSEA between a 186-gene signature prognostic (73 poor prognosis-correlated and 113 good prognosis-correlated genes) in human liver cirrhosis and HCC from mixed etiologies and the pattern of gene expression in DIAMOND mice at 52 weeks NES, normalized enrichment score; FDR, false discovery rate.

The hepatic gene expression at a late time point (week 52) was also different from CD NW-fed mice for the same duration (Fig. 5A–D). In contrast to the changes seen at 8 weeks, there was increased transcriptional activation of oxidative stress, innate immune system and inflammatory pathways at 52 weeks (Fig. 5A–D). This is consistent with data on these pathways in disease progression in humans with NASH [20,21]. Gene ontology (GO) and process networks analysis also identified activation of multiple inflammatory processes with disease progression (Fig. 5A, C). While the metabolic pathway changes noted at 8 weeks were still altered at 52 weeks, they were less prominent.

The hepatic transcriptome of the B6/129 mice was compared to an existing data set of human controls and non-cirrhotic NASH (41 normal/healthy obese and 18 NASH patients) [22]. A strong concordance of the mouse gene expression patterns at 8 and 52 weeks to the human NASH transcriptome was observed (FDR 0.02 and 0.08, respectively) (Fig. 5E). When compared to human cirrhosis of varied etiologies including NASH (186-gene signature, including 73 poor prognosis-correlated and 113 good prognosis-correlated genes) [23,24], the pattern of gene expression within the mouse transcriptome at 52 weeks demonstrated significant concordance with cirrhosis of poor prognosis (FDR <0.001) (Fig. 5F).

### Development of HCC in DIAMOND mice

**Fig. 6:**
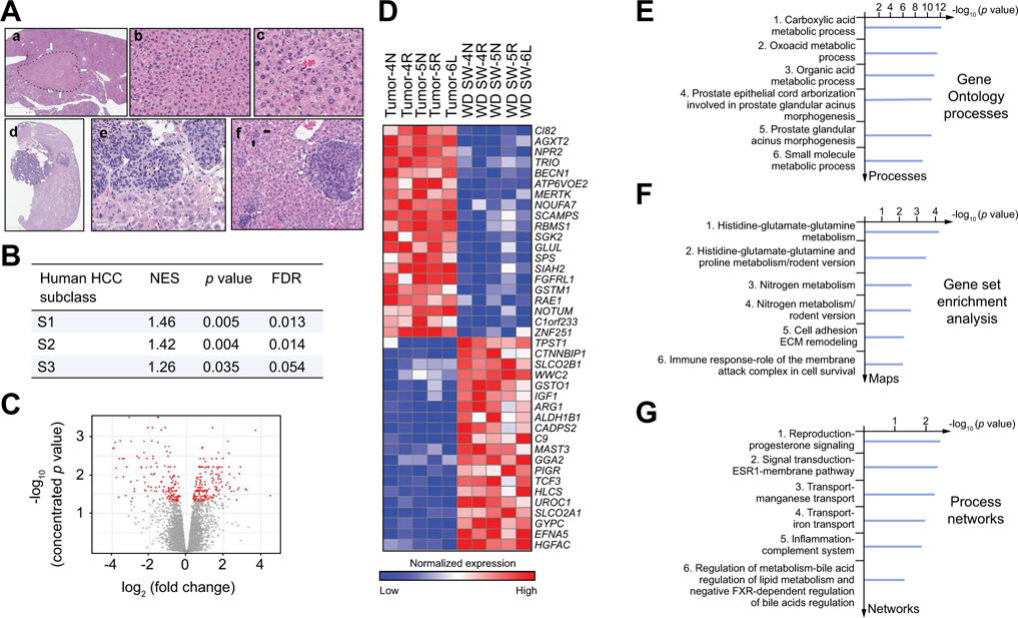
Tumors gene signature in DIAMOND mice at 52 weeks (A) Microscopic views of adenomas (a, b, c) and hepatocarcinomas (HCC) tumors (d, e, f) from B6/129 mice fed a high fructose/glucose, high fat Western diet (WD SW) for 52 weeks. (a) adenoma, (hematoxylin-eosin (H&E), original magnification, ×2.5); (b) cords of hepatocytes with mild atypia and trabecular organization (H&E, original magnification, ×20); (c) unpaired artery between hepatocytes with mild anisocaryosis (H&E, original magnification, ×40); (d) a basophilic well-demarcated tumor with a satellite nodule and with steatosis in the background liver (H&E, original magnification, ×2); (e) interface between malignant tumor (top half) and non-tumoral liver. The lobules of tumoral cells show marked anisocaryosis, eosinophilic cytoplasm, irregular basophilic nuclei and loss of sinusoidal architecture (H&E, original magnification, ×40); (f) satellite nodules made of clusters of tumoral cells (white arrow) and dysplastic foci with multinucleated irregular hepatocytes (black arrow) (H&E, original magnification, ×20). (B) Concordance by gene set enrichment analysis (GSEA) between the gene signatures of human HCC subclasses S1, S2, and S3 and WD SW-induced HCCs in mice at 52 weeks. NES, normalized enrichment score; FDR, false discovery rate. (C–G) Transcriptome analysis was performed on liver adjacent to tumors (WD SW) or tumor tissue (HCC) from WD SW mice at 52 weeks (n = 5). (C) volcano plot; (D) Heat map demonstrating deregulated genes. Red and blue colors indicate high and low gene expression, respectively; (E) Gene ontology (GO) processes; (F) Gene set Enrichment Analysis (GSEA); and (G) Process Networks analysis. The top rank ordered processes, maps and networks are based on statistical significance.

HCC developed in 89% of mice between weeks 32–52. There were 3 or more foci of tumors in each mouse (Figs. 2A, 6A and Supplementary Table 4). All mice had foci of well-differentiated HCC and 40% had poorly differentiated HCC. Also, hepatic adenomas (Supplementary Fig. 6), some with foci of HCC within them, were noted in 25% of the mice. At a transcriptomic level, the HCC transcriptome was related to the S1 or S2 subclasses of human HCC [23] (FDR: 0.013 and 0.014 respectively) (Fig. 6B). Compared to surrounding non-tumorous liver in mice on WD SW, the HCC transcriptome demonstrated activation of several pathways related to nitrogen and amino acid metabolism, oxidative stress signaling, inflammation and cell adhesionextracellular matrix remodeling (Fig. 6C–G). Functional process network analysis (Fig. 6G and Supplementary Fig. 7) revealed changes related to progesterone signaling, bile acid regulation of lipid metabolism, hypoxia and oxidative stress, signal transduction-ESR1 and modulation of apoptosis induced by external signals by estrogen.

### The B6/129 strain is distinct from the parent strains with respect to recapitulating the NAFLD phenotype

**Fig. 7:**
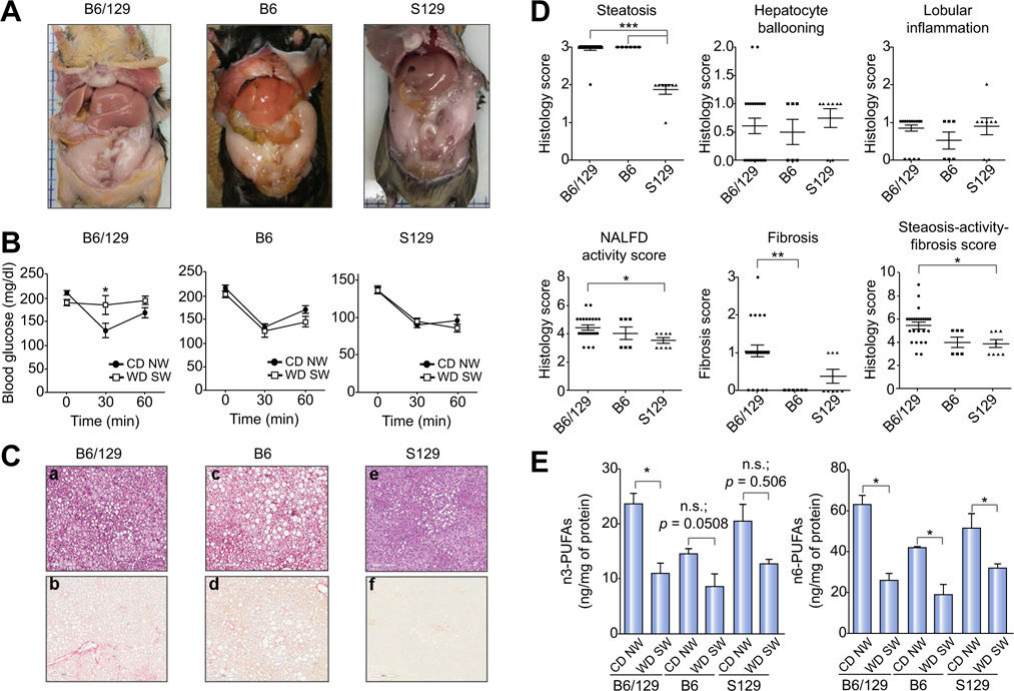
Only the B6/129 background mice, but not the parent strains C57BL/6J (B6) or 129 S1/SvImJ (S129) fed a high fructose/glucose, high fat Western diet (WD SW) for 16–22 weeks depicts the full phenotypic, biological and histological parameters associated with human NAFLD C57BL/6J (B6), 129 S1/SvImJ (S129) or B6/129 mice were fed for 16–22 weeks either a chow diet (CD NW) or a high fructose/sucrose, high fat Western diet (WD SW). (A) Gross liver pictures of WD SW-fed mice for 16–22 weeks, (B) insulin tolerance test (ITT), (C) representative liver sections stained with hematoxylin-eosin (H&E) (a, c, e) or Picrosirius Red (b, d, f) are shown (original magnification, ×10), (D) Histology score for steatosis, hepatocyte ballooning, lobular inflammation, NAFLD activity score, fibrosis and steatosis-activity-fibrosis score. Data represent mean ±SEM for 4–6 mice per CD NW group and 7–13 mice per WD SW group; ***p<0.001, **p <0.01, *p<0.05 WD SW B6/129 vs. WD SW B6 or WD SW S129. (E) Liver n3- and n6-polyunsaturated fatty acids (PUFAs) were measured from B6/129 mice fed a chow diet (CD NW) or high fructose/glucose, high fat Western Diet (WD SW) for 8,16–24 and 52 weeks as described in the Supplementary materials and methods section. Data represent mean ± SEM for 4 mice per group; *p <0.05 WD SW *vs*. CD NW.

Pure 129S1/SvImJ (S129) or C57BL/6J (B6) mice were fed WD SW diet for 16–22 weeks and compared to the B6/129 mice (Fig. 7A). In contrast to the B6/129 mice, both S129 and B6 mice fed WD SW for 16–22 weeks remained insulin sensitive with a marked drop in glucose levels following insulin administration similar to CD NW-fed mice (Fig. 7B). Also, the S129 mice had less hepatomegaly and significantly lower LDL-c and total cholesterol levels compared to B6/129 mice on the combination diet (Fig. 7A and Supplementary Table 5).

Although WD SW feeding induced similar lobular inflammation and hepatocyte ballooning in all three mouse strains at 16–22 weeks, steatosis was lower in S129 mice and there was a trend for decreased fibrosis as well (Fig. 7D). As a consequence, WD SW-fed S129 had a significantly lower NAS and Steatosis-Activity-Fibrosis Score (SAF). The pure B6 mice developed extensive macrovesicular steatosis by 22 weeks, but significantly less fibrosis as compared to B6/129 (Fig. 7C, D). While a longer duration of feeding up to 52 weeks induced greater fibrosis in the pure B6 mice, none of these mice developed any tumors (Supplementary Fig. 8). These results are consistent with the concept that B6/129 strain are more insulin resistant, develop faster steatohepatitis and HCC compared to the B6 mice. These changes occurred despite a similar decrease in n3- and n6-polyunsaturated fatty acids in all three strains (Fig. 7E).

To further gain insights into the differences between the B6 *vs*. B6/129 mice, their full hepatic transcriptomes on WD SW were compared in an unbiased manner at week 52. The B6/129 mice had significantly greater activation of multiple pathways that are known to be relevant for both NASH and oncogenesis. Cell adhesion/cell matrix interactions, regulation of angiogenesis, ECM remodeling, unfolded protein response, insulin signaling and bile acid regulation of lipid metabolism related networks were all upregulated in B6/129 mice compared to the B6 strain (Supplementary Figs. 9 and 10). Interestingly, both collagen IV-osteopontin-FAK1 network (Supplementary Table 6), as well as an activin A-MMP-2-syntenin 2 network, both of which have been associated with HCC development in humans [25–29], were upregulated in B6/129 mice as compared to B6 after 52 weeks of WD SW diet. Collectively, these results indicate that the crossed mouse background B6/129 fed a WD SW constitute a faster and more powerful model than the pure B6 mouse to recreate all biochemical and histological parameters and HCC development as seen in human NASH.

## Discussion

Animal models of human disease are an important element of translational science required to better model the disease at a molecular level, identify targets for therapeutics and for preclinical testing of specific compounds before embarking on expensive human clinical trials. To meet these needs, such models should come as close to human disease as possible. In the context of NASH, models should display all of the physiological, metabolic, histological and clinical endpoints of human NASH.

We here describe a mouse model (DIAMOND) where fatty liver disease was induced by caloric excess as occurs in most humans with NASH. Upon initiation of WD SW, the mice gained weight, became insulin resistant and developed dyslipidemia. They also sequentially developed steatosis, steatohepatitis and increasing fibrosis progressing to bridging fibrosis. It is remarkable that, given the challenges in recapitulating humanlike steatohepatitis in mice, the mice developed florid steatohepatitis with ballooning and Mallory-Denk bodies based on *a priori* criteria using the FLIP algorithm [30]. The mice also developed spontaneous HCC without the need for additional manipulations. At a transcriptomic level, there was a concordance in terms of the pathways activated in humans with NAFLD and mice with corresponding histologic phenotypes. Several key protein pathways relevant to human NASH were also activated in these mice. These mice therefore are similar to human disease in terms of initiation with caloric excess, changes in metabolic status and lipid abnormalities, liver histology, development of progressive fibrosis and HCC, changes in signaling pathways and activation of pathways at a transcriptomic level.

Also, this model is distinct from both parent strains. The 129S1/SvImJ (S129) mice failed to develop both insulin resistance and clear cut steatohepatitis. While the C57BL/6J (B6) mice eventually develop insulin resistance, they take a longer time to develop this hallmark of the metabolic syndrome. Also, their histology is milder and they do not develop HCC. At a molecular level, there was a substantially greater activation of cell stress and oncogenic pathways relevant for human disease in the B6/129 mice. The genetic basis for this predilection for HCC is an important question and future studies may provide some novel information on HCC development following prolonged lipotoxic stress.

While there were many similarities between the DIAMOND model and human NASH, it is also recognized that there were some dissimilarities. A large proportion of the mice developed spontaneous HCC while it is known that only a small proportion of afflicted humans develop HCC [31]. As with most other mice models, this model does not develop significant atherosclerosis as seen in obese humans with NAFLD [31]. Also, fully established cirrhosis was not commonly seen by week 52. It is possible that further dietary manipulation or knocking in the mutant human *PNPLA3* gene may accelerate fibrosis but remains a topic of future studies.

We anticipate that the DIAMOND mice could serve as a tool to study several scientific questions in the future. These include NASH, hepatic fibrosis and potentially HCC. The observed decrease in steatosis despite maintained insulin resistance in advanced disease recapitulates what is seen in humans and sets the stage to study this phenomenon mechanistically. It is unlikely to be due to weight loss, which appears to coincide clinically with onset of HCC, because the animals remain insulin resistant. Also, the development of bridging fibrosis in this model renders it more relevant model to study NASH-related fibrosis rather than traditional bile duct ligation or thioacetamide models. This model may additionally serve as another tool to understand the genetics of NASH especially with in depth analysis of the parent strains and the crossed strain in our model. Changes in the microbiome, as observed in human disease, remained to be evaluated in the DIAMOND model to further permit the study of dietmicrobiome interactions and their relationship to NASH. The authors look forward to future scientific endeavors and collaborations to tackle these interesting questions.

It is important to note that this model was not meant to and nor does it provide a novel unique mechanism for disease development that is distinct from all existing models. Rather, it recapitulates the physiological, metabolic, histological aspects of human NASH in the same model along with strong concordance with human disease with respect to changes within the transcriptome and activation of pathways known to be relevant for NASH.

In summary, this DIAMOND recapitulates the various phenotypes of NAFLD and their associated metabolic and underlying molecular characteristics. It is hoped that it will serve as a relevant model to identify therapeutic targets, model disease progression and test preventive and therapeutic approaches against NASH, hepatic fibrosis and HCC.

## Supplementary Material

Supplemental Material

## Abbreviations

ALT: alanine aminotransferase
AST: aspartate aminotransferase
B6: C57BL/6J
CD: chow diet
CK-18: cytokeratin-18
DIAMOND: diet-induced animal model of non-alcoholic fatty liver disease
ER: endoplasmic reticulum
FDR: false discovery rate
GTT: glucose tolerance test
IRE-1: inositol-requiring enzyme-1
ITT: insulin tolerance test
JNK: c-Jun NH2-terminal kinase
LDL-c: low-density lipoprotein-cholesterol
MCD: methionine choline deficient
NAFLD: non-alcoholic fatty liver disease
NASH: non-alcoholic steatohepatitis
NW: normal water
PERK: protein kinase R-like endoplasmic reticulum kinase
PUFA: polyunsaturated fatty acid
T2DM: type 2 diabetes mellitus
S129: 129S1/SvImJ
HCC: hepatocellular carcinoma
WD: Western diet
SW: sugar water
