# Supplementary material for: A diet-induced animal model of non-alcoholic fatty liver disease and hepatocellular cancer
Source: J Hepatol. Author manuscript; Available in PMC 2016 Sep 6. (PMC5012902; doi:10.1016/j.jhep.2016.05.005)
Supplement: Supplemental Material [file NIHMS812241-supplement-Supplemental_Material.pdf]

# A diet-induced animal model of non-alcoholic fatty liver disease and hepatocellular cancer

Amon Asgharpour, Sophie C. Cazanave, Tommy Pacana, Mulugeta Seneshaw, Robert Vincent, Bubu A. Banini, Divya Prasanna Kumar, Kalyani Daita, Hae-Ki Min, Faridoddin Mirshahi, Pierre Bedossa, Xiaochen Sun, Yujin Hoshida, Srinivas V. Koduru, Daniel Contaifer Jr., Urszula Osinska Warncke, Dayanjan S. Wijesinghe, Arun J. Sanyal

## Table of contents

**Supplementary materials and methods…………………………………………..….2**
**Supplementary Fig. 1…………………………………………………………………14**
**Supplementary Fig. 2…………………………………………………………………15**
**Supplementary Fig. 3…………………………………………………………………17**
**Supplementary Fig. 4…………………………………………………………………19**
**Supplementary Fig. 5…………………………………………………………………21**
**Supplementary Fig. 6…………………………………………………………………25**
**Supplementary Fig. 7…………………………………………………………………26**
**Supplementary Fig. 8…………………………………………………………………30**
**Supplementary Fig. 9…………………………………………………………………31**
**Supplementary Fig. 10………………………………………………………………33**
**Supplementary Table 1………………………………………………………………37**
**Supplementary Table 2………………………………………………………………38**
**Supplementary Table 3………………………………………………………………39**
**Supplementary Table 4………………………………………………………………41**
**Supplementary Table 5………………………………………………………………42**
**Supplementary Table 6………………………………………………………………43**
**Supplementary references…………………………………………………………45**

## Supplementary materials and methods

**B6/129 mice background.** B6/129 were initially a gift from Dr Sandra Erickson of University of California San Francisco, CA and derived from mice with C57BL/6J and 129S1/SvImJ backgrounds. The strain was started by mating the F2, second filial, generation (Jackson Stock # 101045) with one another. With progressive generations derived from brother-sister mating, the hepatic phenotype of NAFLD developed with increasing consistency following initiation of a high fat and carbohydrate diet. After 20 generations derived from brother-sister mating, an isogenic strain was developed from the original F2 strain of mice.

**Glucose and Insulin Tolerance Tests (GTT, ITT).** Mice were fasted overnight and baseline blood glucose levels were measured in tail-vein blood using an Accu-Chek Compact plus glucometer (Roche Diagnostics). Glucose (1 mg dextrose/g body weight) in sterile phosphate buffered saline was injected intraperitoneally, and blood glucose levels were measured prior to, and 15, 30, 60, 90 and 120 min after glucose injection using established protocols [1]. For the insulin tolerance test, mice were fasted for 4 h and intraperitoneally injected with regular insulin (0.75 U/kg body weight) using established methodology [1]. Blood glucose levels were measured prior to, and 30 and 60 min after insulin injection. GTT and ITT were performed several days from each other on the same mice.

**Histological Analysis.** The presence of steatosis and type of steatosis (micro- and macrovesicular) as well as its distribution were noted. Steatohepatitis was defined by the presence of steatosis, inflammation and hepatocellular ballooning [2, 3], as per the FLIP algorithm [4]. The severity of steatosis, lobular inflammation and hepatocellular ballooning were scored using the NASH-Clinical Research Network (CRN) criteria [5].

Specifically, the amount of steatosis (percentage of hepatocytes containing fat droplets) was scored as 0 (<5%), 1 (5–33%), 2 (>33–66%) and 3 (>66%). Hepatocyte ballooning was classified as 0 (none), 1 (few) or 2 (many cells/prominent ballooning). Foci of lobular inflammation were scored as 0 (no foci), 1 (<2 foci per 200× field), 2 (2–4 foci per 200× field) and 3 (>4 foci per 200× field). The NAFLD activity score (NAS) was computed from the grade of steatosis, inflammation and ballooning. Fibrosis was scored as stage F0 (no fibrosis), stage F1a (mild, zone 3, perisinusoidal fibrosis), stage F1b (moderate, zone 3, perisinusoidal fibrosis), stage F1c (portal/periportal fibrosis), stage F2 (perisinusoidal and portal/periportal fibrosis), stage F3 (bridging fibrosis) and stage F4 (cirrhosis). Quantitative analysis of fibrosis was performed by morphometry from digitalized Sirius Red-stained sections using the Aperio system after tuning the threshold of fibrosis detection under visual control. For ease, F1a, F1b, and F1c fibrosis were all scored as 1. The results were expressed as the percent collagen proportional area (CPA) as previously published [6]. Human NASH liver samples used to compare main histological features with DIAMOND mice belong to an archive of human NASH samples scored as previously described [5]. Histological classification of tumor was based on usual criteria. Adenoma was defined as a nodular proliferation of normal hepatocytes retaining a trabecular organization, well-differentiated HCC include cytological and architectural abnormalities such as major trabecula thickening, loss of sinusoidal barrier, pseudo-acinar formation and isolated arteries. Poorly differentiated HCC were characterized by major cytological abnormalities such as significant increase in nuclear/cytoplasmic ratio, architectural disorganization with loss of trabecular organization.

**Liver Sample Collection and Processing.** Mice were weighed and then exposed to inhaled isoflurane prior to being euthanized. Euthanasia was performed by cervical

dislocation, and a laparotomy was performed with visual inspection of all abdominal organs. The liver was then removed *in toto* from the abdominal cavity and weighed. The appearance of the liver surface and its color was noted. The number of visible tumors, defined by gross masses apparent at the liver surface with or without hemorrhage, and their size were counted. The liver was then sectioned in a sagittal plane and the number of tumors within the liver counted. A portion of the liver was then placed in separately labeled containers of 10% formalin (VWR) for later histologic processing and analysis. When tumors were present, they were dissected from the remaining liver and were placed into separate cryotubes and snap frozen in liquid nitrogen. For histologic analysis of tumors, masses were taken with surrounding non-tumor parenchymal tissue and placed in containers of 10% formalin. The remaining liver was aliquoted into several cryotubes that either contained RNA Later (Ambion, Life Technologies) or were simply snap-frozen in liquid nitrogen.

**Blood Collection and Analysis.** Blood was collected via retro-orbital bleeding using heparinized micro-hematocrit capillary tubes (Fisher Technology) into untreated Eppendorf tubes and centrifuged for 15 min at 1,500 g at 4°C. The serum was then collected and stored at -80°C for later analysis.

Alanine aminotransferase (ALT) and aspartate aminotransferase (AST) were measured in serum samples using an Advia 1800 Chemistry System (Siemens). Both ALT and AST measurements utilize the Alanine Aminotransferase (ALTP5P)/ Aspartate Aminotransferase (ASTP5P) method respectively using pyridoxal-5'-phosphate and the addition of α−ketoglutarate [7]. The concentration of NADH is measured by its absorbance at 340/410 nm. The decrease in absorbance values reflects utilization of NADH and is thus proportional to AST or ALT activity in their respective reactions.

Lipid analysis including determination of triglycerides, cholesterol, and calculated low-density lipoprotein levels was performed in serum samples also using the Advia 1800 Chemistry System (Siemens). For triglyceride measurement the Triglycerides (TRIG) method was based on the Fossati three-step enzymatic reaction with a Trinder endpoint [8]. The single-reagent procedure quantitates the total triglycerides including the mono and diglycerides and the free glycerol fractions. The method for cholesterol measurement was based on the determination of Δ4-cholestenone after enzymatic cleavage of the cholesterol ester by cholesterol esterase, conversion of cholesterol by cholesterol oxidase, and subsequent measurement by the Trinder reaction of the hydrogen peroxide formed [9]. The calculated low-density lipoprotein was determined by subtracting the determined high-density lipoprotein and one-fifth of the triglycerides measured from the total cholesterol [10]. Serum adiponectin was measured using mouse Adiponectin/Acrp30 Quantikine ELISA Kit from R&D Systems (Minneapolis, MN).

**Immunoblot Analysis.** Tissue lysates were prepared as previously described [11]. Equal amounts of protein (30 µg) were resolved by SDS-PAGE on a 4%-12% NuPAGE Novex Bis-Tris Mini Gels (Invitrogen), then transferred to nitrocellulose membranes, and incubated with primary antibodies. Membranes were incubated with appropriate horseradish peroxidase-conjugated secondary antibodies (Cell Signaling Technology). Bound antibody was visualized using the SuperSignal chemiluminescent kit (Thermo Scientific Pierce Biotechnology) and the chemiluminescent signal was detected using the FluorChem E chemiluminescence system (ProteinSimple). Antibodies used were obtained from the following sources: rabbit anti-PUMA (sc-28226), rabbit anti-phospho PERK (Thr 981) (sc-32577), rabbit anti-PERK (sc-13073) (Santa Cruz Biotechnology, Inc.); rabbit anti-JNK (#9252), rabbit anti-phospho-JNK (Thr183/Thr185) (#9251), rabbit

anti-PARP (#9532), rabbit anti-Caspase 3 (#9665), rabbit anti-Bim (#2819), rabbit anti-p42/p44 (4377), rabbit anti-phospho-p42/p44 (#4695), rabbit anti-Fatty acid synthase (#3189), rabbit anti-Acetyl-CoA Carboxylase (#3662) and rabbit anti-phospho-Acetyl-CoA Carboxylase (Ser79) (#3662) (Cell Signaling Technology). β-actin-HRP (ab49900) was purchased from Abcam.

**Desmin, α-SMA and CK-18 Immunostaining.** Staining was performed for desmin, which is a marker of hepatic stellate cells (HSC) and alpha-smooth muscle actin (α-SMA), which is a marker of activated HSC. Liver specimens embedded in paraffin blocks were used. Three µm sections from each block were stained with either rabbit anti-desmin (Abcam, ab15200), α-SMA (Abcam, ab5694) or CK-8/18 (Dako, clone EP17/EP30) at appropriate dilutions. Staining was performed on a VENTANA DISCOVERY immunohistochemical platform (Roche Diagnostic) using OmniMap anti-Rabbit HRP as a conjugate and ChromoMap DAB as a chromogen (Roche Diagnostic). Semi-quantitation of α-SMA-positive nucleated cells was performed on 20 high power field (HPF), and graded as 0: no or rare positive cells/HPF, 1: <5 positive cells/HPF, 2: 5 to 10 positive cells/HPF, 3: >10 positive cells/HPF often in continuous layer.

**RNA Isolation.** Liver tissues lysed in RLT lysis buffer (QIAGEN, 74104) were shipped to the QIAGEN Service Core for Genomics and Gene Expression. RNA was isolated using RNEasy Mini Kit (QIAGEN, 74104) following the manufacturer's protocol. RNA quality was determined using the Agilent Bioanalyzer (Agilent) with RNA 6000 Nano Kits (Agilent, 5067-1511). Total RNA yield, 260/280, and 260/230 ratios were measured using a NanoDrop spectrophotometer (Thermo).

**Quantitative Real-Time Polymerase Chain Reaction.** Total RNA was reverse-transcribed into complementary DNA with Moloney leukemia virus reverse transcriptase

and random primers (both from Invitrogen). Quantification of the complementary DNA template was performed by real-time PCR using SYBR green fluorescence on a LightCycler 480 instrument (Roche Applied Science). Primers were as followed: mouse CHOP (NM_007837): forward 5'- GCAGTCATGGCAGCTGAGTC-3' and reverse 5'-CGCAGGGTCAAGAGTAGTGA-3'; mouse GAPDH (NM_008084.2): forward 5'-AGAAACCTGCCAAGTATGATG-3' and reverse 5'-GGAGTTGCTGTTGAAGTCG-3'. The relative abundance of mRNA was expressed relative to the amount of the reference gene Glyceraldehyde-3-phosphate dehydrogenase (GAPDH) according to the comparative threshold cycle (Ct) method.

**Xbp-1 splicing.** Total RNA was isolated and spliced Xbp-1 (Xbp-1s) and unspliced Xbp-1 (Xbp-1u) were detected by RT-PCR using the following primers: forward: 5'-AAACAGAGTAGCAGCGCAGACTGC-3' and reverse 5'-TCCTTCTGGGTAGACCTCTGGGAG-3', then followed by digestion by the restriction enzyme Pst-I, as previously described [12].

**Lipid Analytical Approaches.** Preparation of samples for analysis: Liver samples were subjected to homogenization to uniformity in ice cold PBS such that a final suspension of 10% w/v liver particles in PBS were obtained.

For TAG measurement, 50 µL of liver homogenate was diluted with 150 µL of PBS. To the mixture thus obtained, 20 µL of deuterated TAG internal standard (IS) mixture (LM-6000, Avanti Polar Lipids, Alabaster, AL containing 4 µM each of 20:5-22:6-20:5 D5 TG, 14:0-16:1-14:0 D5 TG, 15:0-18:1-15:0 D5 TG, 16:0-18:0-16:0 D5 TG, 17:0-17:1-17:0 D5 TG, 19:0-12:0-19:0 D5 TG, 20:0-20:1-20:0 D5 TG, 20:2-18:3-20:2 D5 TG, 20:4-18:3-20:4 D5 TG) was added. The mixture thus obtained was vortexed and kept on ice for 10

minutes for equilibration of the IS with the sample. Following incubation, 1 ml of methanol was added to the sample followed by sonication to mix the samples. Thereafter 0.5 ml of chloroform was added and the samples were sonicated again to mix the solvents to obtain a monophasic extraction system and to disperse any clumps. The mixture thus obtained was incubated at 48°C for one hour. Following incubation, a phase break was carried out by the addition of 1 mL of chloroform followed by 2 mL of water. The resultant mixture was vortexed for 30 seconds and then centrifuged at 3,500 x g to enable phase separation. Following centrifugation the bottom organic layer containing the lipids was transferred to a fresh tube. The top aqueous layer was washed with an additional 1 mL of $CHCl_3$, vortexed, centrifuged and the separated organic layer was combined with the previous organic fraction. The lipid extract thus obtained was dried via vacuum centrifugation and re-suspended in 200 µL of methanol. The re-suspended solution was incubated at 48°C for 10 minutes vortexed, and 50 µL transferred to 200 µL autosampler vials for analysis via mass spectrometry. TAG's analysis was performed by mass spectrometry using 5 µL of the lipid extract prepared as above and analyzed following separation on a XSelect HSS-T3 C18 reverse phase column (100Å, 3.5 µm, 3 mm X 150 mm, Waters) using a Shimadzu Nexera UPLC chromatographic system coupled to Sciex TripleTOF 5600+ quadrupole time of flight mass analyzer. Data was acquired in the positive mode in an untargeted data dependent manner in the mass range of 50-1200 Da. A 28 minute chromatographic separation utilizing a linear gradient of 60:40 acetonitrile:water with 10mM ammonium formate and 0.1% formic acid as mobile phase A and 90:10 isopropanol:acetonitrile with 10mM ammonium formate and 0.1% formic acid as mobile phase B. Following analysis the data was interrogated for TAG's using LipidViewTM software and the raw intensity counts were converted to quantitative units

via comparison against those for the known amounts of internal standards. The final data was supplied as pmols of the TAG/mg of protein.

For FFA measurement, to 200 µL of liver homogenate a 20 µL of a deuterated internal standard mixture was added containing 10 ng each of (d8) Arachidonic acid, (d6) dihomo gamma linoleic acid, (d5) docosahexaenoic acid, (d5) eicosapentaenoic acid, (d8) 5-hydroxyeicosatetranoic acid (5 HETE), (d8) 12-hydroxyeicosatetranoic acid (12 HETE), (d8) 15-hydroxyeicosatetranoic acid (15 HETE), (d8) 20-hydroxyeicosatetranoic acid (20 HETE). The mixture was vortexed and allowed to come to equilibrium by incubating on ice for 10 minutes. Lipids were extracted from this mixture via the addition of 1ml of ice cold ethanol followed be sonication to disperse the particles and incubation overnight at -200°C. The following morning the samples were vortexed and centrifuged at 15,000 x g to remove particulate material. The clarified supernatant was harvested, dried via vacuum centrifugation and re-suspended in 100 µL of 50:50 ethanol:water. Free fatty acids and their oxygenated products were quantitatively analyzed via UPLC – ESI MS/MS as described previously xx. Ten µL of the lipid extract reconstituted in 50:50 ethanol:water (100 µL) were subjected to analysis via UPLC ESI-MS/MS. A 14 minute reversed-phase LC method utilizing a Kinetex C18 reverse phase column (100 x 2.1mm, 1.7µm) and a Shimadzu Nexera UPLC was used to separate the eicosanoids at a flow rate of 500 µL/min at 50°C. The column was first equilibrated with 100% Solvent A [acetonitrile:water:acetic acid (20:80:0.02, v/v/v)] for two minutes and then 10 µL of sample was injected. 100% Solvent A was used for the first two minutes of elution. Solvent B [acetonitrile:isopropanol (20:80, v/v)] was increased in a linear gradient to 25% Solvent B to 3 minutes, to 30% by 6 minutes, to 55% by 6.1 minutes, to 70% by 10 minutes, and

to 100% by 10.1 minutes. 100% Solvent B was held until 13 minutes, then was decreased to 0% by 13.1 minutes and held at 0% until 14 minutes. The eluting eicosanoids were analyzed using a hybrid triple quadrupole linear ion trap mass analyzer (Sciex 6500 QTRAP®,) via multiple-reaction monitoring (MRM) in negative-ion mode. Eicosanoids were monitored using species specific precursor → product MRM pairs. The mass spectrometer parameters used were: curtain gas: 30; CAD: High; ion spray voltage: -3,500V; temperature: 500°C; Gas 1: 40; Gas 2: 60; declustering potential, collision energy, and cell exit potential were optimized per transition.

**Microarray Analysis.** Biotin-aRNA was generated using The Epicentre TargetAmp-Nano Labeling Kit for Illumina Expression BeadChip (Cat # TAN07908) from an input of 300 ng of high quality RNA with RIN number >8.0. Illumina bead arrays were done using mouse WG6 Expression BeadChip kits (Illumina) (Cat #BD-201-0602) according to manufacturer's protocol. Raw intensity values were acquired using the HiScan microarray scanner and imported to GenomeStudio using the Gene Expression Module (both from Illumina). Raw data-sets without normalization or background correction on the arrays were exported from GenomeStudio (Illumina) to a Genespring (Agilent) formatted file. Intensity values were normalized to the median of all samples. Fold change and statistical analysis was done using volcano plot filtering with a fold change of 2 and greater, and $p<0.05$.

**Bioinformatic Analysis.** Microarray data was imported into Genespring GX 12.5 for transcriptome/gene expression analysis. RMA algorithm was employed on the raw data for normalization followed by validations of the data-sets. Quantile normalization tests were also performed as part of this step. In addition, baseline transformation, where the median of the log-summarized values were calculated and subtracted from each of the

samples, was performed to bring the median of all samples to the same value without affecting downstream analyses. Quality control was performed using 3-dimensional principal component analysis (PCA) plot method. Samples from each experimental condition clustered together validating the high-quality of the data-sets. Correlation plots were also performed with calculation of Pearson's correlation coefficient and visualized as heatmaps to further confirm concordance of gene expression from one sample to another within each experimental group.

Following quality control tests, a two-sample t-test coupled with false discovery rate (FDR) correction via Benjamini-Hochberg method was employed to compare differences in the whole transcriptome gene expression between the experimental groups and the control samples as previously described [13]. A threshold of fold-change of 2.0 and $p<0.05$ was employed to identify/detect significantly differentially-modulated genes between the experimental and controls groups.

Next, to determine the functional relevance of the significantly modulated gene lists, we performed gene-set enrichment analysis (GSEA) and gene ontology (GO) analysis. The GO Term Mapper (GOTM) database includes vocabularies for description of molecular function, biologic processes and cellular components of gene products. The GO terms have a directed acrylic graph (DAG) structure that ensures that a gene with a particular GO term also has several other ancestor GO terms implicitly. GO terms were generated via Genespring GX 12.5 using Benjamini-Yekutieli FDR correction method to identify significantly modulated gene modules between experimental and control groups. Enrichment analysis was performed for MetaCore's curated pathways as previously described[14].

Pathway analysis of differentially expressed genes was performed using the

MetaCore pathway analysis suite (Thomson Reuters, New York, NY)[14] for functional gene networks. Differentially regulated gene lists with $p<0.05$ and fold change of 2.0 were used from individual groups as input for the MetaCore pathway analysis software and differentially regulated pathways maps were generated using the pathway enrichment analysis workflow on the MetaCore platform.

To identify the additional functionally relevant genes and gene-targets modulated between the experimental and control groups, gene set enrichment analysis (GSEA) was also performed using the Molecular Signatures Database (MSigDB) (>10,000 gene sets)[15]: signaling pathways (217 sets, BioCarta), metabolic pathways (186 sets, KEGG), microRNA target genes (221 sets), transcription factor targets (615 sets), target gene signatures for chemical and genetic perturbations in literature (3,402 sets), gene ontology (1,454 sets, GO), global curated pathway gene sets (674 sets, REACTOME), and target gene signatures of oncogenic signaling in literature (189 sets). A normalized enrichment score (NES) was used to evaluate concordance between the mouse hepatic gene signature and a poor prognostic human NASH hepatic NASH with cirrhosis gene signature data set and human hepatocellular carcinomas[16-18]. Similarity of global transcriptome of our model to human NASH [19] [NCBI, Gene Expression Omnibus accession number GSE48452] was assessed using Subclass Mapping algorithm[20] implemented in GenePattern genomic analysis toolkit (www.broadinstitute.org/genepattern). Transcriptome datasets of our mouse models are available at NCBI Gene Expression Omnibus database (www.ncbi.nlm.nih.gov/geo) with accession number GSE67680.

**Statistical Analysis.** Descriptive statistics were used to describe the distribution of laboratory and histological findings using Excel and Prizm version 5.0. All data are

expressed as the mean ± S.E. of the mean. Inter-group comparisons were made using analysis of variance (ANOVA) with *post hoc* Bonferroni correction for multiple comparisons as appropriate for normally distributed variables. The statistical analysis plan for bioinformatics analyses are noted in supplemental materials. A two-tailed *p* value of 0.05 was set to establish statistical significance.

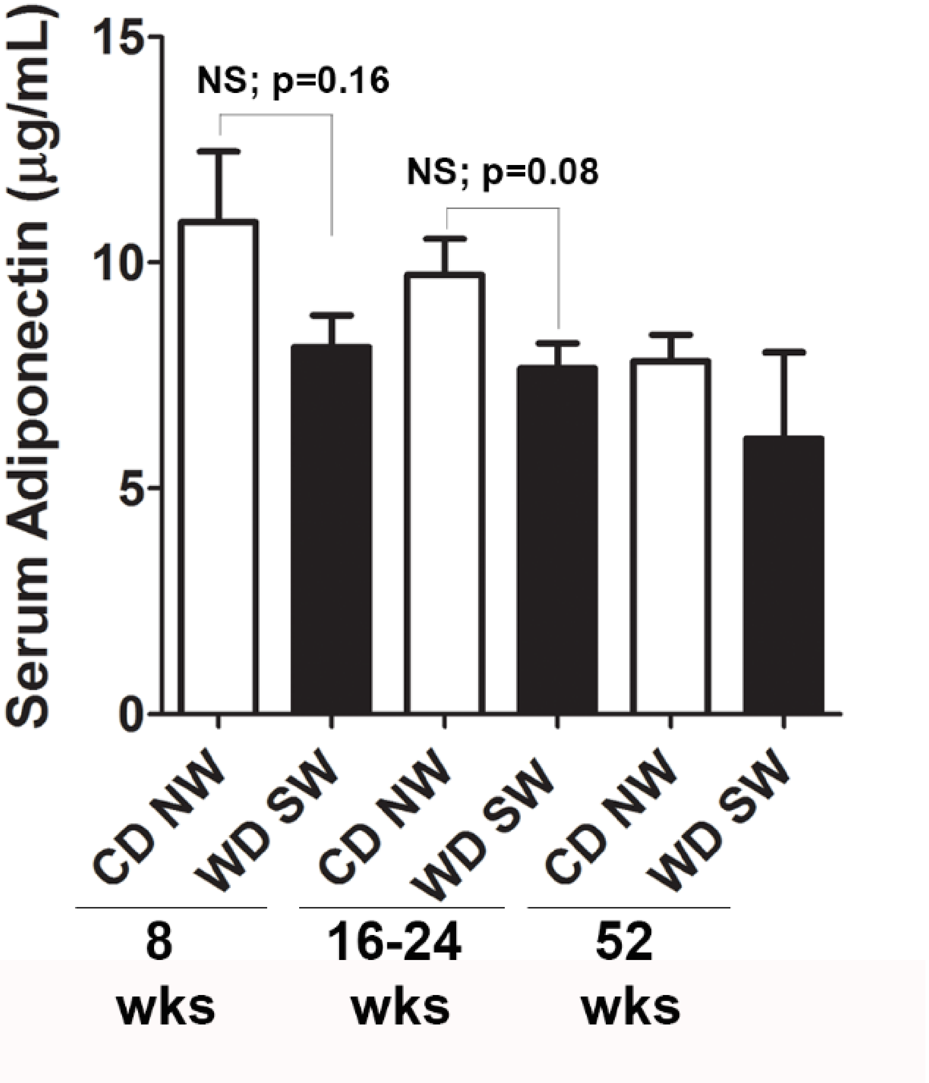


**Supplementary Fig. 1. Serum adiponectin levels in mice fed a high fructose/glucose, high fat Western diet (WD SW).** Serum adiponectin levels were measured from B6/129 mice fed a chow diet (CD NW) or high fructose/glucose, high fat Western Diet (WD SW) for 8, 16-24 and 52 weeks. Data represent mean ± SEM for 4 mice per group.

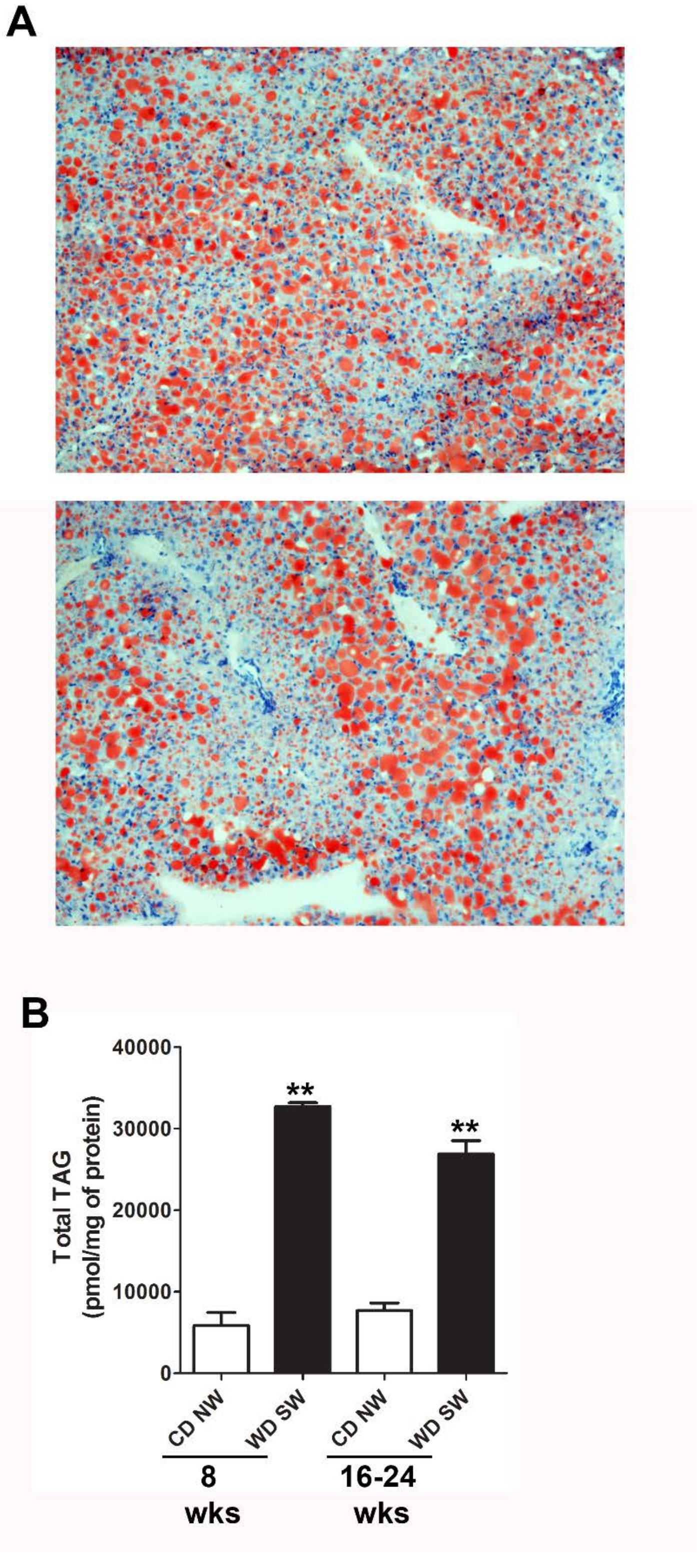
A
B
40000
30000
20000
10000
0
Total TAG
(pmol/mg of protein)
**
**
CD NW
WD SW
CD NW
WD SW
8
wks
16-24
wks

**Supplementary Fig. 2. Hepatic steatosis in mice fed a high fructose/glucose, high fat Western diet (WD SW).** (A) Representative Oil red O–stained liver cryostat sections from livers from WD SW mice at 8 weeks of diet. Original magnification, ×10. (B) Total liver triglycerides (TAG) were measured from B6/129 mice fed a chow diet (CD NW) or high fructose/glucose, high fat Western Diet (WD SW) for 8 and 16-24 weeks. Data represent mean ± SEM for 4 mice per group; **$P < 0.01$ WD SW versus CD NW.

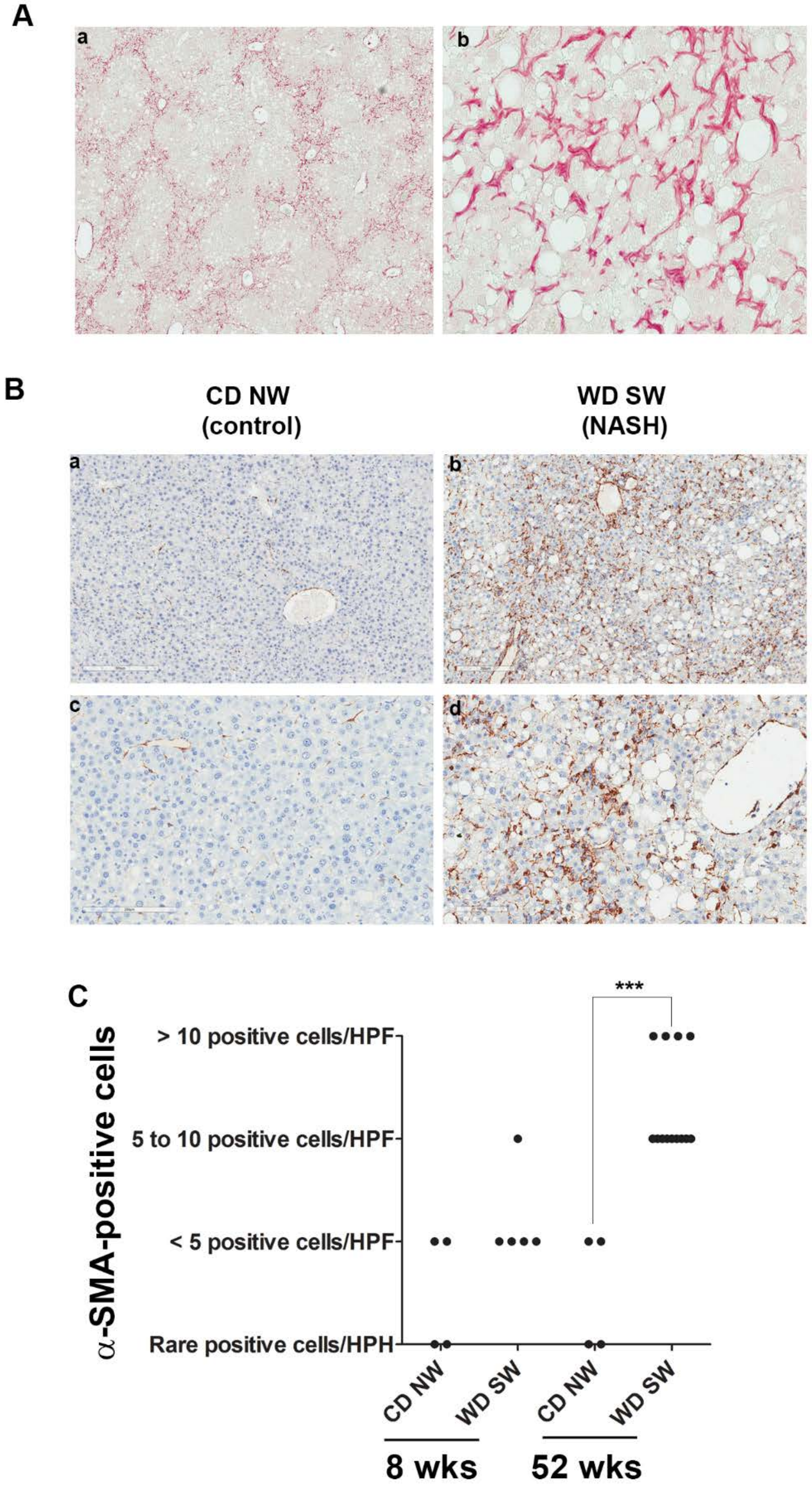


**Supplementary Fig. 3. Pericellular fibrosis and activated hepatic stellate cells are observed in B6/129 mice fed a high fat Western diet (WD SW) for 52 weeks.** (A)

Pericellular fibrosis induced by a high fructose/glucose, high fat Western diet (WD SW) for 52 weeks. (a) Sirius Red staining showing extensive fibrosis with bridging (Original magnification, ×5); (b) Fibrosis is mainly composed of collagen fibers laying between rows of hepatocytes with steatosis (Sirius Red staining, original magnification, ×40) (B) Representative images of liver tissue from mice fed a chow diet (CD NW) (a,c) or high fructose/glucose, high fat Western Diet (WD SW) (b,d) for 52 weeks stained with desmin. Original magnification, ×10 (a-b) and ×20 (c-d). (C) Semi-quantitation of α-SMA-positive cells in liver tissue from mice fed a CD NW or WD SW for 8 and 52 weeks. HPF, high power field. Data represent mean ± SEM for 4-13 mice per group; ****$P < 0.001$* WD SW versus CD NW.

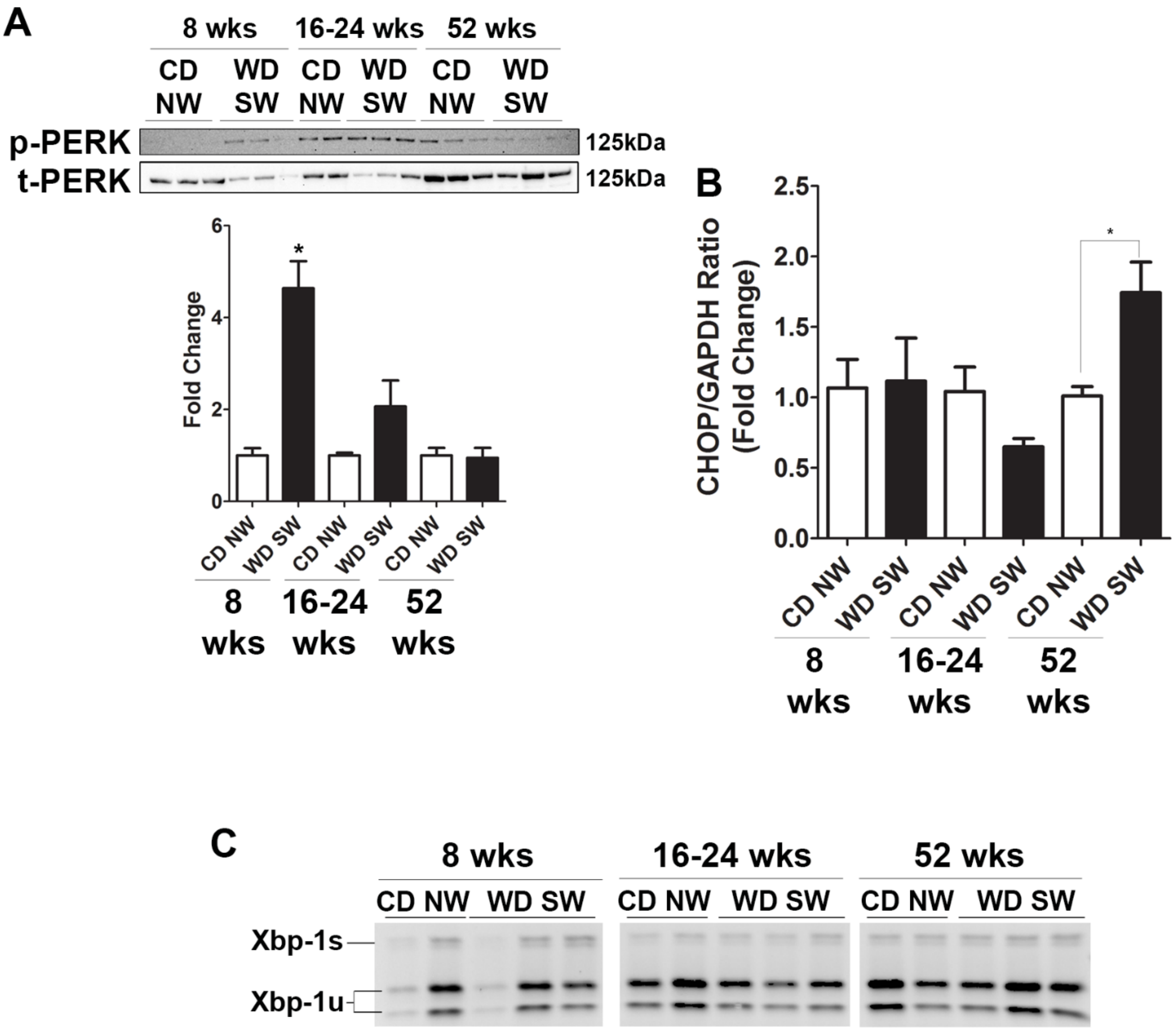


**Supplementary Fig. 4. Endoplasmic reticulum (ER) stress responses are activated in B6/129 mice fed a high fat Western diet (WD SW).** Total RNA and whole cell lysates were prepared from liver tissue from B6/129 mice fed a chow diet (CD NW) or high fructose/glucose, high fat Western Diet (WD SW) for 8, 16-24 or 52 weeks. (A) Immunoblot analysis were performed for phosphorylated and total PERK (p-PERK and t-PERK). p-PERK/t-PERK ratio were calculated by densitometry and normalized to CD NW-fed mice. (B) *CHOP* mRNA expression was profiled by real-time PCR. Fold change

is relative to internal control GAPDH. (C) Unspliced and spliced forms of *Xbp-1* mRNA (Xbp-1u and Xbp-1s) were assessed using a PCR-based assay as described in "Supplementary Materials and Methods" section. Data represent mean ± SEM for 4-5 mice per group; **P* < *0.05* WD SW versus CD NW.

A

# Blood coagulation

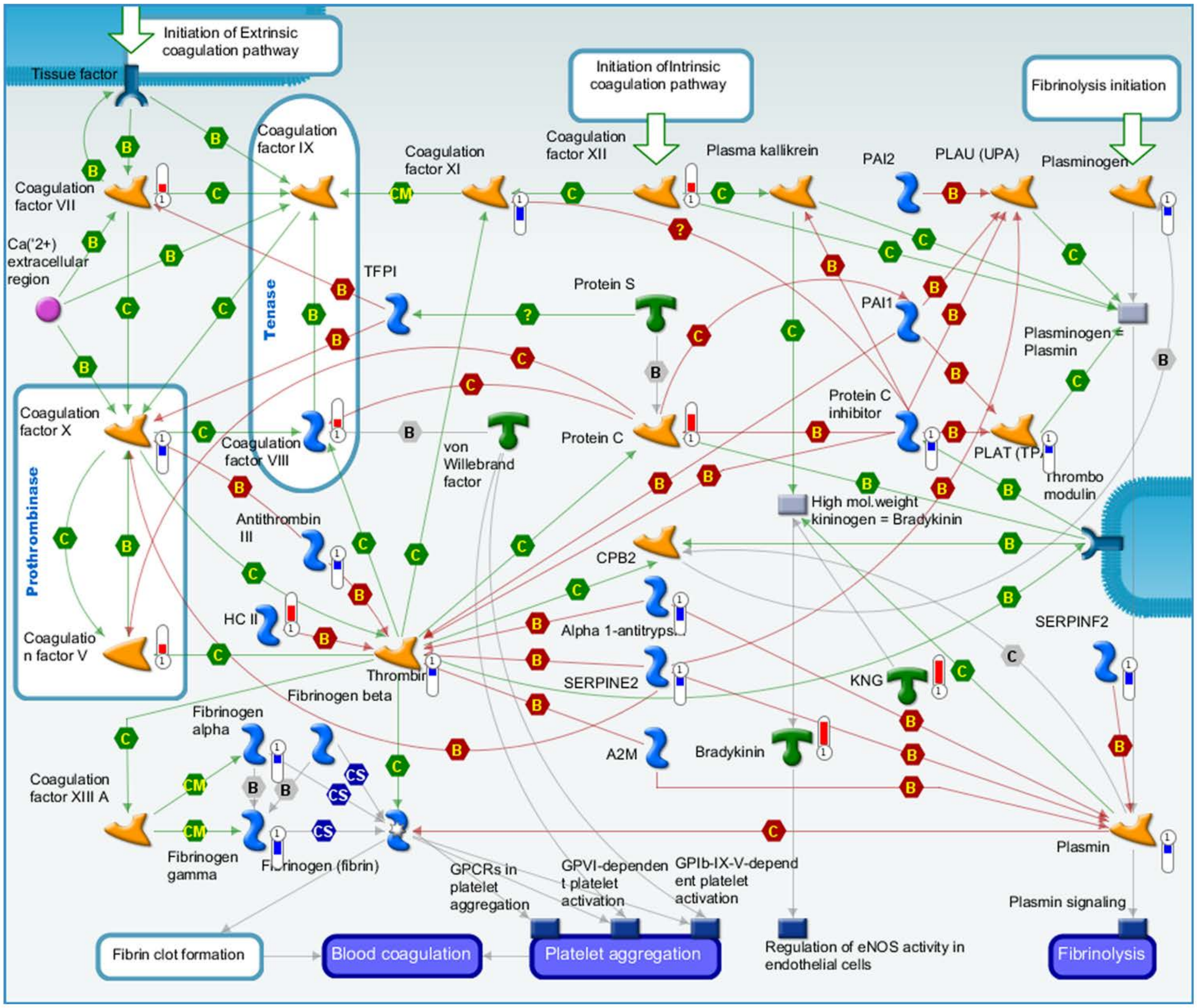

**B**

# Cytoskeleton remodeling
# TGF, WNT and cytoskeletal remodeling

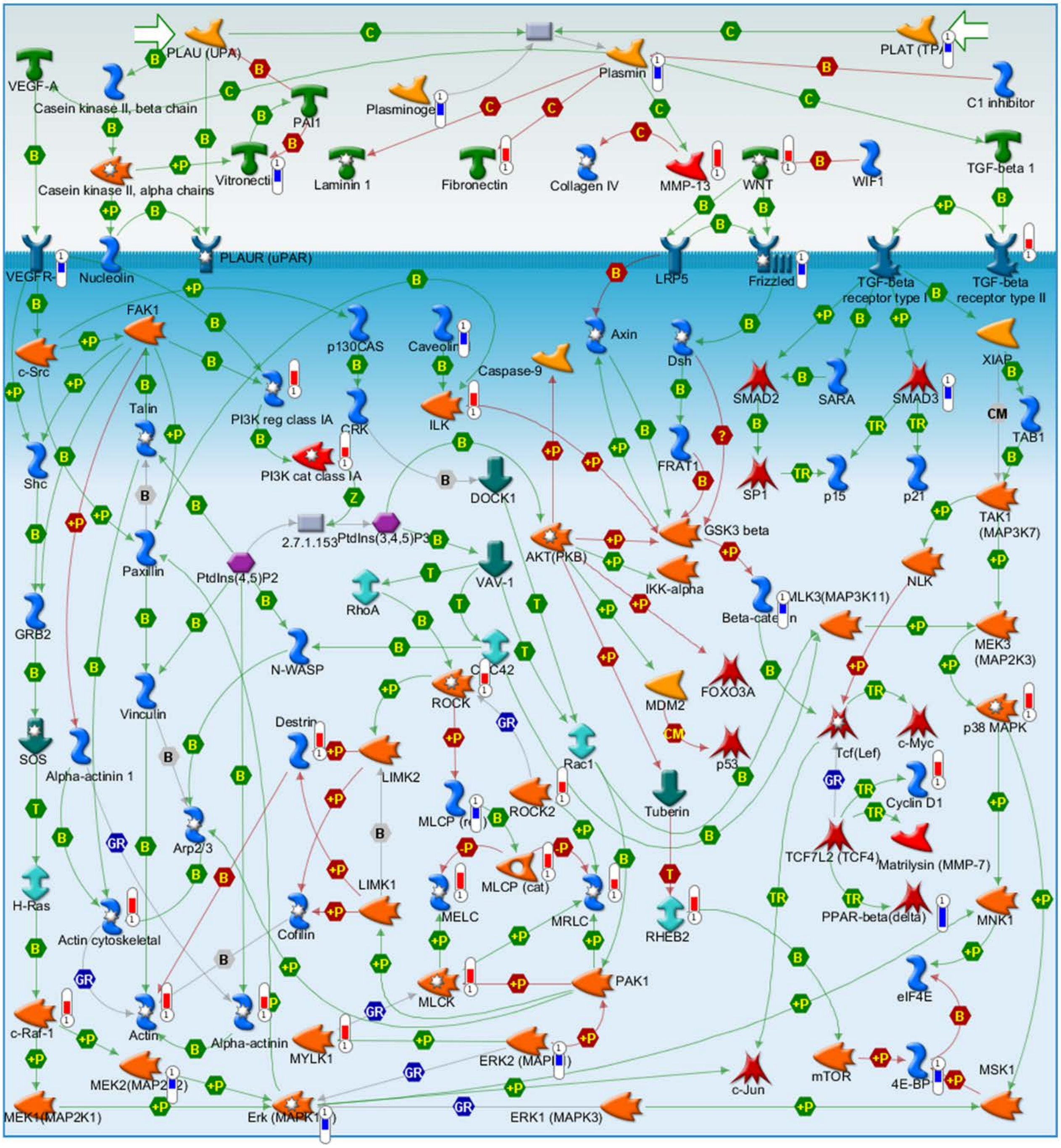

C

# LRRK2 in neurons in Parkinson's disease

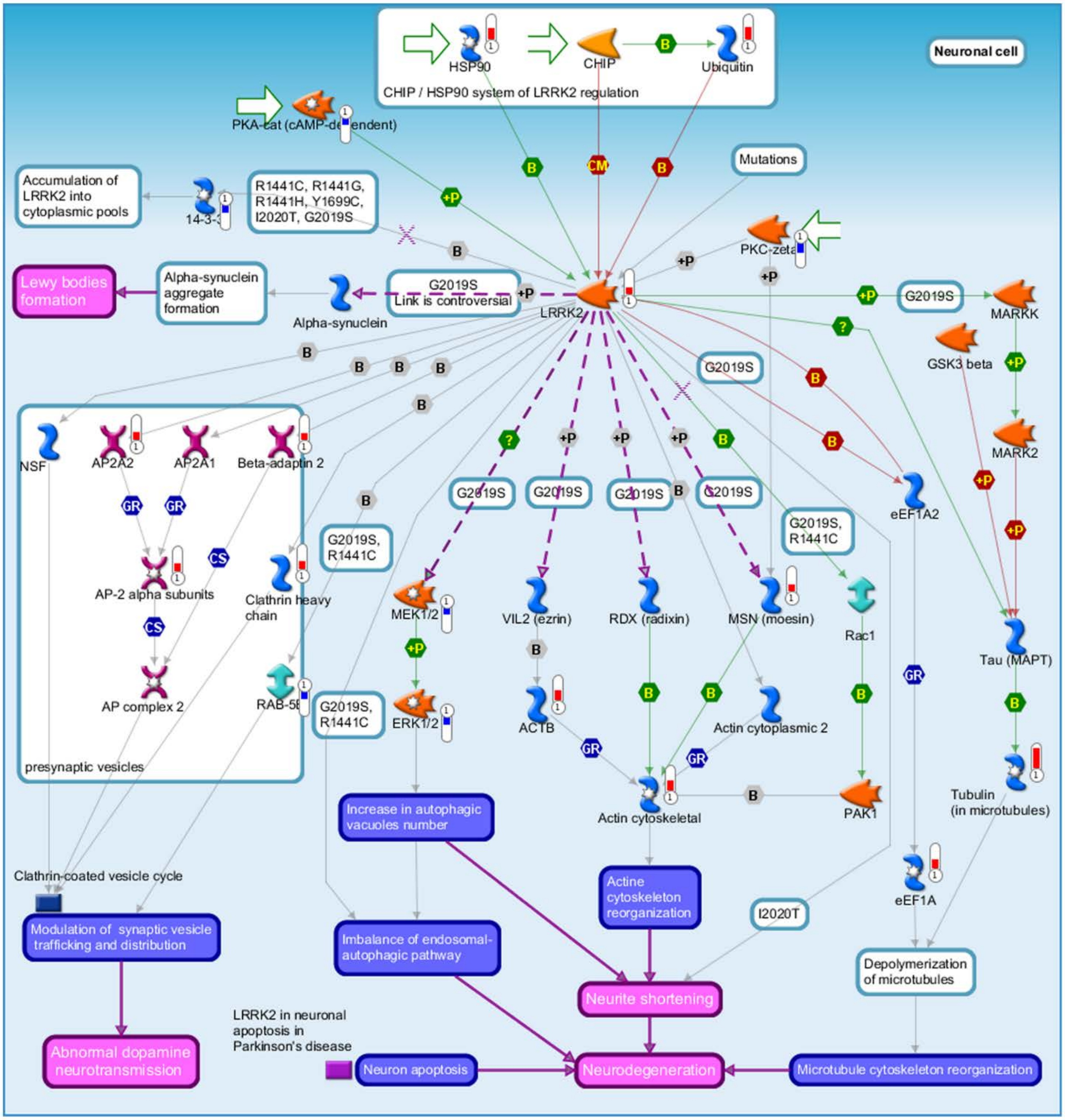


**Supplementary Fig. 5. Top rank Gene Set Enrichment Analysis based on statistical significance in mice fed a high fructose/glucose, high fat Western diet (WD SW) for**

**8 weeks.** (A) Blood coagulation pathway. (B) Cytoskeleton remodeling-TGF, WNT and cytoskeletal remodeling. (C) LRRK2 in neurons in Parkinson's disease. Pathways maps were visualized using the MetaCore pathway analysis suite (Thomson Reuters, New York, NY). Experimental data from all files is linked to and visualized on the maps as thermometer-like figures. Up-ward thermometers have red color and indicate up-regulated signals and down-ward (blue) ones indicate down-regulated expression levels of the genes.

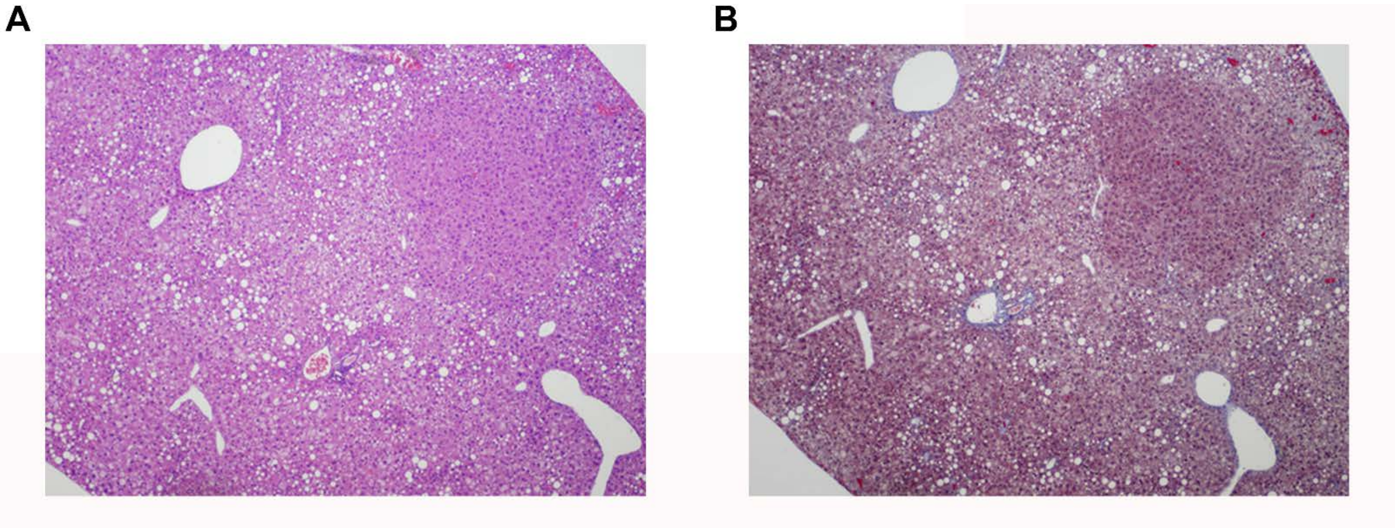


**Supplementary Fig. 6. Hepatic adenoma in mice fed a high fructose/glucose, high fat Western diet (WD SW) for 52 weeks.** (A-B) Additional microscopic views of livers from WD SW mice at 52 weeks of diet. Liver sections stained with hematoxylin-eosin (A) or trichrome blue (B) depicting a well-delineated benign hepatic adenoma. Original magnification, ×2.5.

**A**

# Androstenedione and testosterone biosynthesis and metabolism p.1/ Rodent version

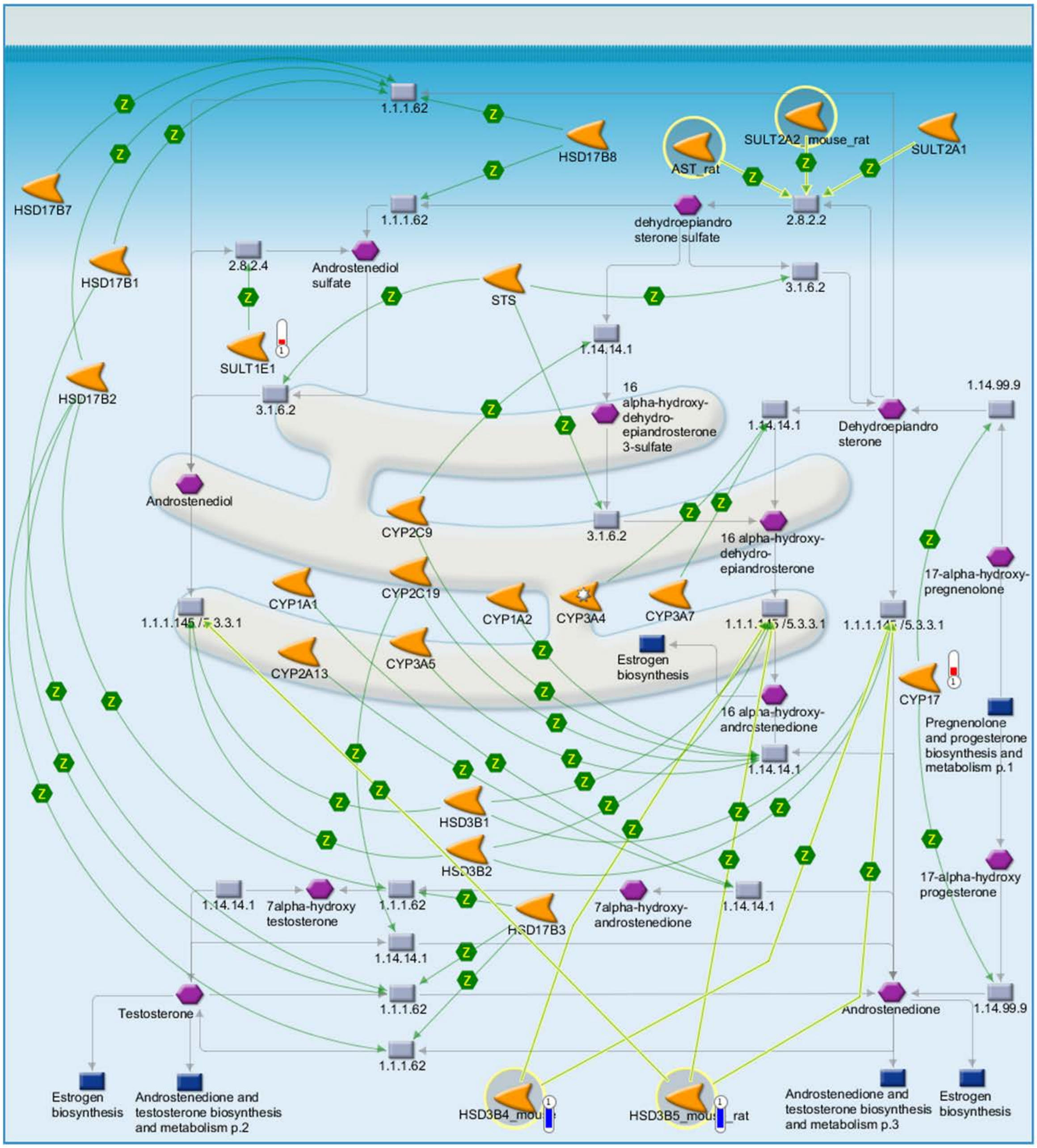

**B**

# Role of ZNF202 in regulation of expression of genes involved in atherosclerosis

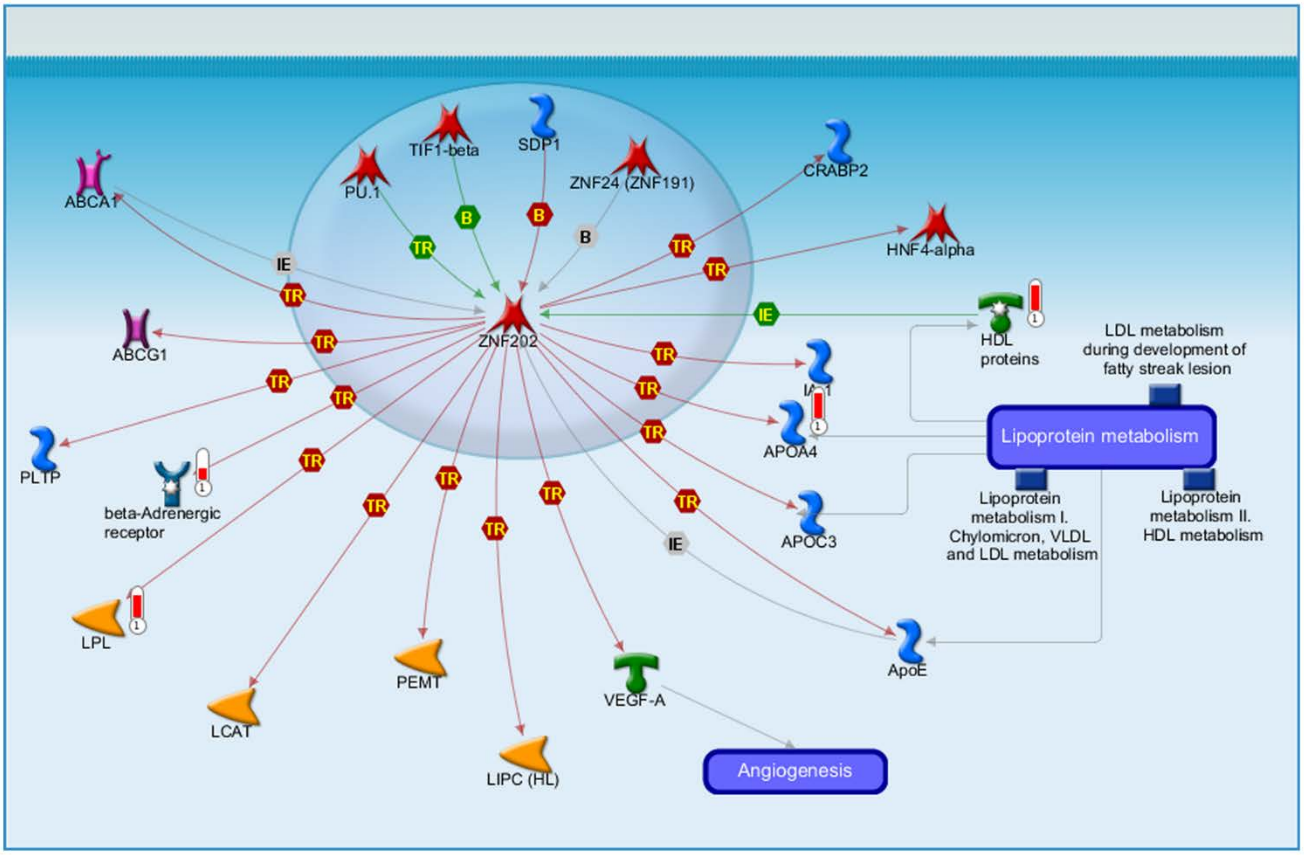

C

**Oxidative stress_Role of IL 8 signaling pathway in respiratory burst**

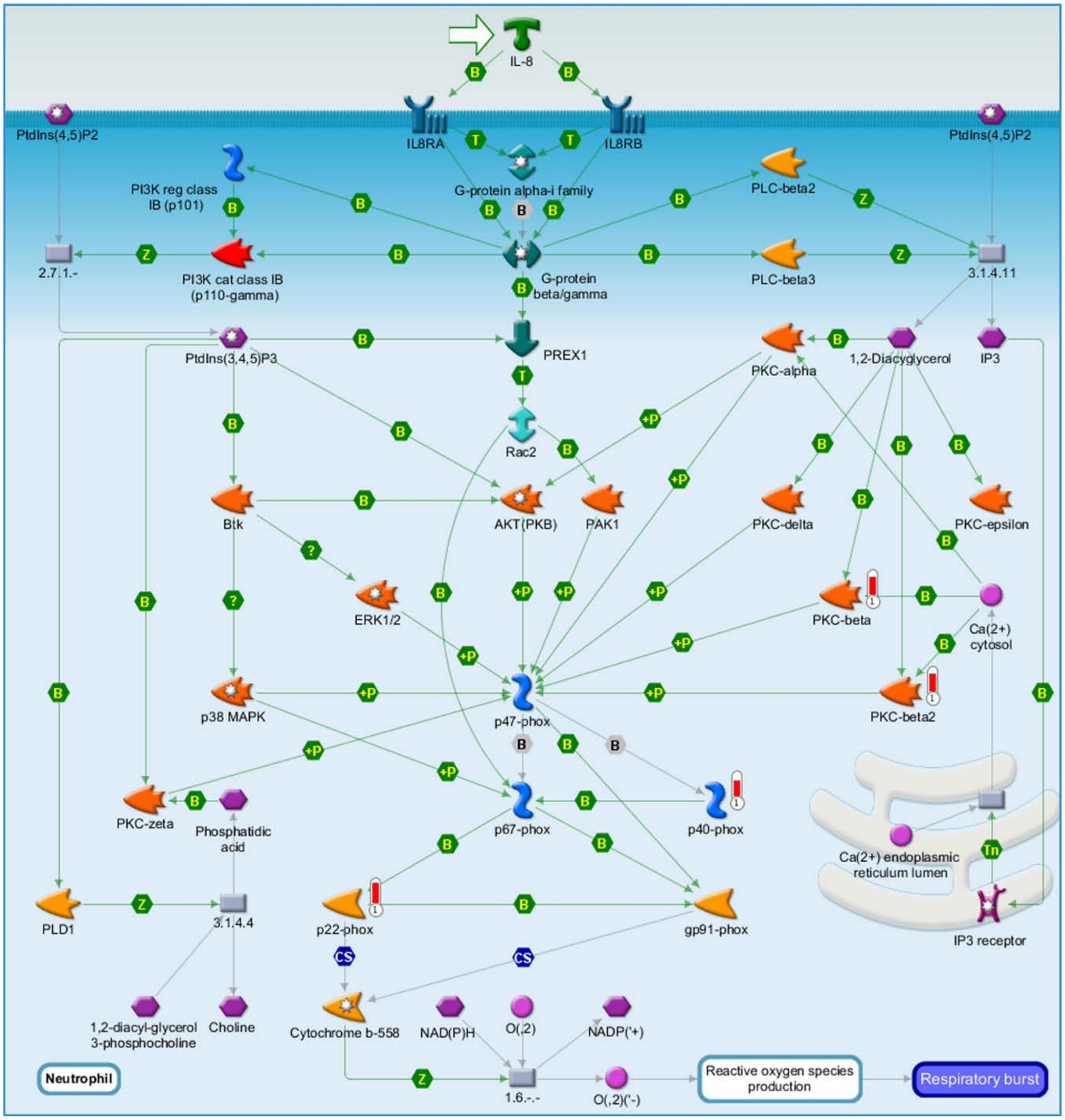


**Supplementary Fig. 7. Top rank Gene Set Enrichment Analysis based on statistical significance in mice fed a high fructose/glucose, high fat Western diet (WD SW) for 52 weeks.** (A) Androstenedione and testosterone biosynthesis and metabolism p.1/rodent version. (B) Role of ZNF202 in regulation of expression of genes involved in

atherosclerosis. (C) Oxydative stress_Role of IL-8 signaling pathway in respiratory burst**.** Pathways maps were visualized using the MetaCore pathway analysis suite (Thomson Reuters, New York, NY). Experimental data from all files is linked to and visualized on the maps as thermometer-like figures. Up-ward thermometers have red color and indicate up-regulated signals and down-ward (blue) ones indicate down-regulated expression levels of the genes.

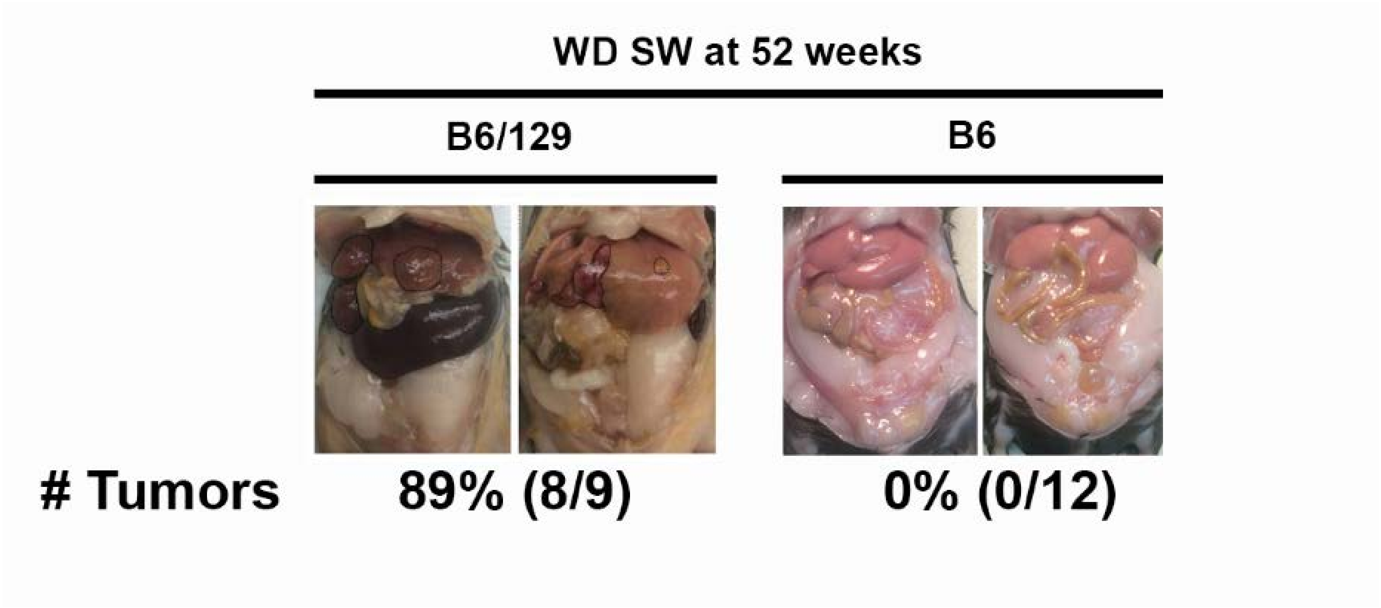


**Supplementary Fig. 8. Pure C57BL/6J (B6) mice fed a high fructose/glucose, high fat Western Diet (WD SW) did not develop tumors at 52 weeks.** Gross livers from B6/129 or pure B6 mice fed a high fructose/glucose, high fat Western Diet (WD SW) for 52 weeks. In B6/129 mice fed a WD SW for 52 weeks, multiple foci of tumors were observed at the time of necropsy in 8 out of a total of 9 mice; in contrast, no pure B6 fed a WD SW diet for 52 weeks had tumors out of a total of 12 mice.

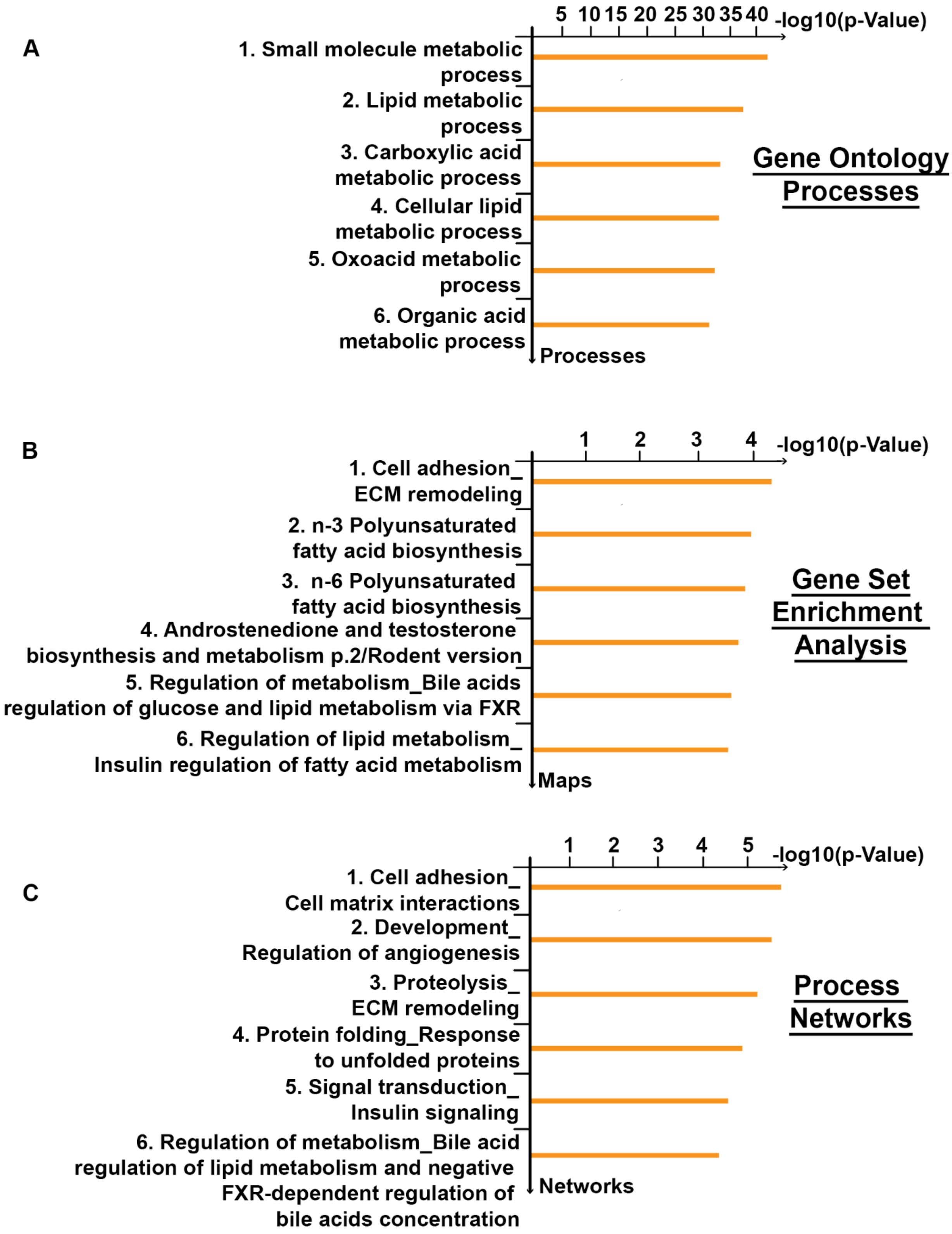
A
5 10 15 20 25 30 35 40 -log10(p-Value)
1. Small molecule metabolic process
2. Lipid metabolic process
3. Carboxylic acid metabolic process
4. Cellular lipid metabolic process
5. Oxoacid metabolic process
6. Organic acid metabolic process
Processes
Gene Ontology Processes
B
1 2 3 4 -log10(p-Value)
1. Cell adhesion_ ECM remodeling
2. n-3 Polyunsaturated fatty acid biosynthesis
3. n-6 Polyunsaturated fatty acid biosynthesis
4. Androstenedione and testosterone biosynthesis and metabolism p.2/Rodent version
5. Regulation of metabolism_Bile acids regulation of glucose and lipid metabolism via FXR
6. Regulation of lipid metabolism_ Insulin regulation of fatty acid metabolism
Maps
Gene Set Enrichment Analysis
C
1 2 3 4 5 -log10(p-Value)
1. Cell adhesion_ Cell matrix interactions
2. Development_ Regulation of angiogenesis
3. Proteolysis_ ECM remodeling
4. Protein folding_Response to unfolded proteins
5. Signal transduction_ Insulin signaling
6. Regulation of metabolism_Bile acid regulation of lipid metabolism and negative FXR-dependent regulation of bile acids concentration
Networks
Process Networks

**Supplementary Fig. 9. Hepatic gene expression dataset in B6/129 mouse fed a high fructose/glucose, high fat Western Diet (WD SW) for 52 weeks as compared to pure C57BL/6J (B6) mice fed WD SW for 52 weeks.** Transcriptome analysis using the Illumina mouse WG6 Expression BeadChip kits (Illumina) was performed on liver tissues from B6/129 or B6 mice fed WD SW for 52 weeks of diet (n=5 per group). The data are presented as: (A) Gene ontology (GO) processes; (B) Gene set Enrichment Analysis (GSEA); and (C) Process Networks analysis. The top rank ordered processes, maps and networks are based on statistical significance.

**A**

# Cell adhesion_ECM remodeling

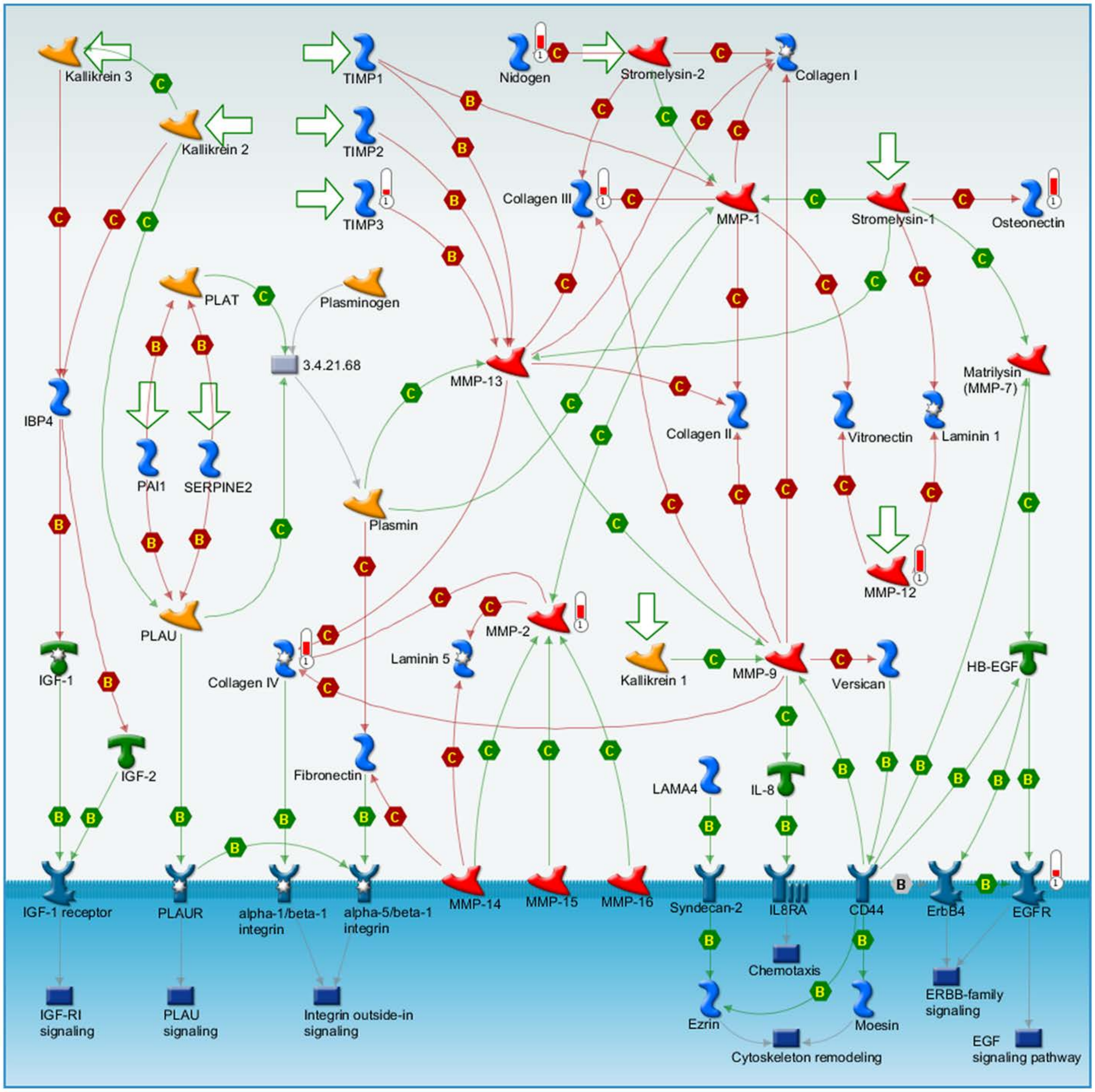

**B** **n-3 Polyunsaturated fatty acid biosynthesis**

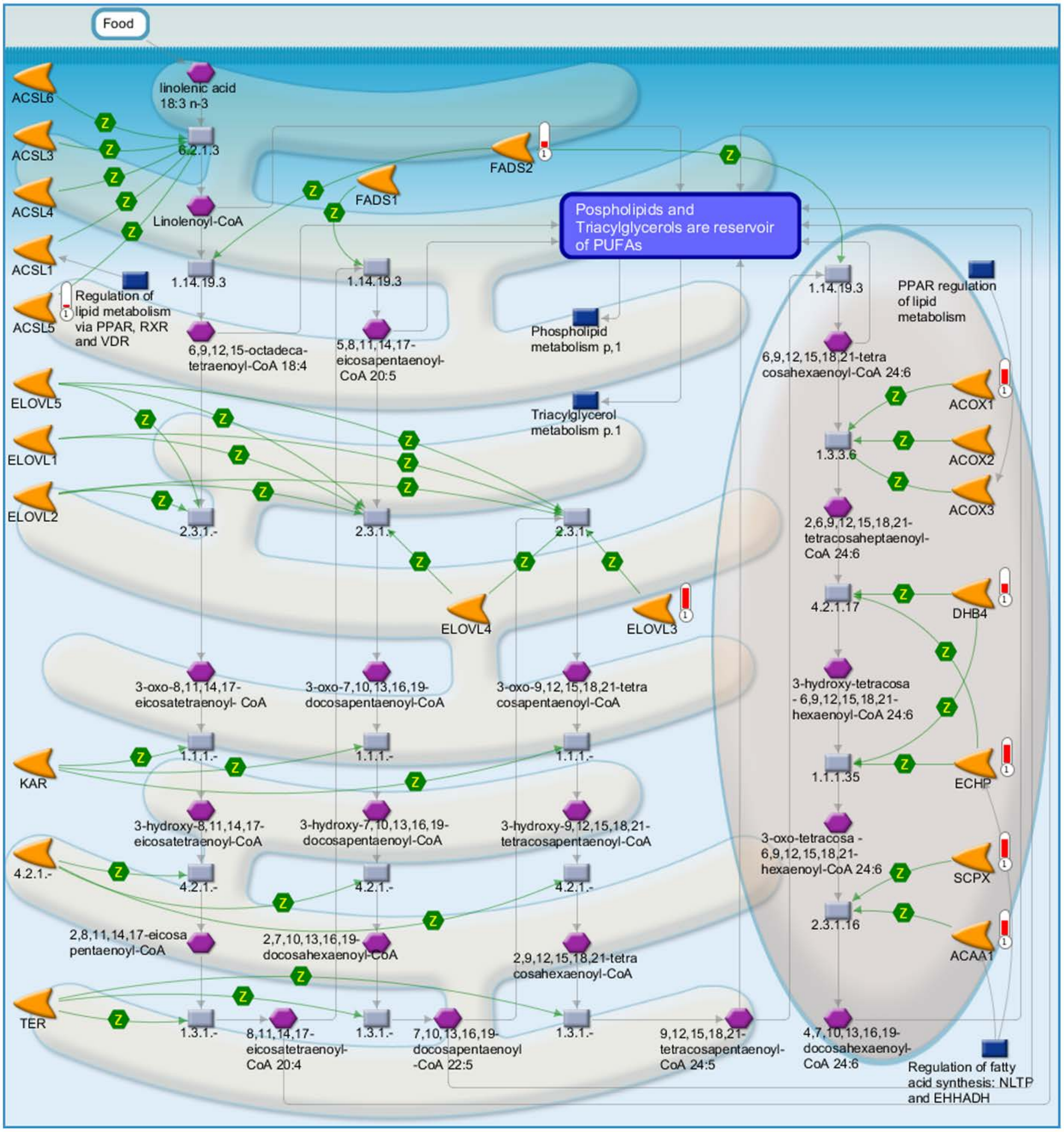

C

## n-6 Polyunsaturated fatty acid biosynthesis

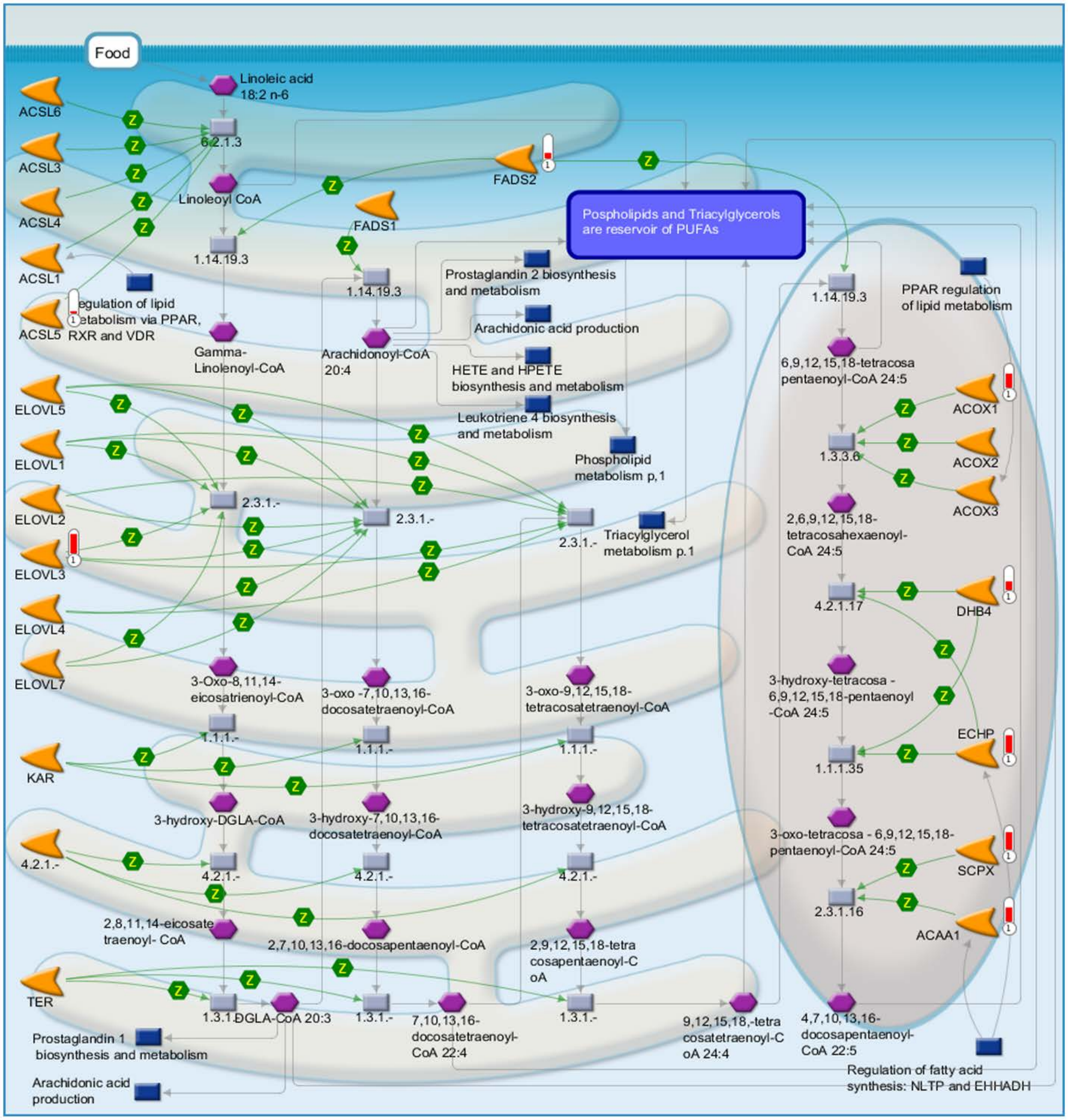


**Supplementary Fig. 10. Top rank Gene Set Enrichment Analysis based on statistical significance in B6/29 mice fed a high fructose/glucose, high fat Western diet (WD SW) for 52 weeks as compared to pure C57BL/6J (B6) mice fed WD SW for**

**52 weeks.** (A) Cell adhesion_ECM remodeling. (B) n-3 Polyunsaturated fatty acid biosynthesis. (C) n-6 Polyunsaturated fatty acid biosynthesis. Pathways maps were visualized using the MetaCore pathway analysis suite (Thomson Reuters, New York, NY). Experimental data from all files is linked to and visualized on the maps as thermometer-like figures. Up-ward thermometers have red color and indicate up-regulated signals and down-ward (blue) ones indicate down-regulated expression levels of the genes.

**Supplementary Table 1. Biochemical and pathological characteristics of animal models of NASH**

| Characteristic | Human [21] | MCD [22] | Ob/Ob [23] | PTEN [24] | STAM [25, 26] | ALIOS [27] | Ossabaw (swine) [28] | HF-HC-HSD [29] * | CD-HFD [30] |
|---|---|---|---|---|---|---|---|---|---|
| **Weight gain** | + | - | + | - | - | +/- | + | + | + |
| **Insulin Resistance** | + | - | + | - | - | +/- | + | + | |
| **Diabetes (type)** | + (T2DM) | - | + (T2DM) | - | + (T1DM) | +/- (T2DM) | + (T2DM) | + (T2D M) | + (T2DM) |
| **hypertriglyceridemia** | + | - | + | - | + | + | + | + | + |
| **Gene knock-out** | - | - | + | + | - | - | - | - | - |
| **Steatosis** | + | + | + | + | + | + | + | + | + |
| **Lobular inflammation** | + | + | - | + | + | + | + | + | +/- |
| **Hepatocyte ballooning** | + | + | - | + | + | - | + | + | + |
| **Mallory-Denk bodies** | + | - | - | + | - | - | - | - | + |
| **Pericellular fibrosis** | + | + | - | - | + | + | + | + | +/- ** |
| **Cirrhosis** | + | - | - | + | + | - | - | - | - |
| **Hepato-cellular Carcinoma** | + | - | - | + | + | + | - | + | + *** |

MCD, methionine and choline deficient; PTEN, phosphatase and tensin homolog; STAM, streptozotocin-induced NASH; ALIOS, American Lifestyle Induced Obesity Syndrome; CD-HFD, HF-HC-HSD, High Fat-High Cholesterol-High Sugar Diet; Choline Deficient-High Fat Diet.

*This diet contains 10% cholesterol, which does not constitute a physiological atherogenic diet (≤ 0.2% cholesterol). **Only mild fibrosis was depicted in CD-HFD mice at 12 months; ***only 25% of CD-HFD mice develop tumors at 12 months.

**Supplementary Table 2. Background strain characterization of the C57BL/6J;129S1/SvImJ (B6/129) mouse strain**

| Chromosome # (# of SNPs analyzed) | SNPs C57BL/6J background (%) | SNPs 129S1/SvImJ background (%) |
|---|---|---|
| **Chromosome 1 (11 SNPs)** | 27.3 | 72.7 |
| **Chromosome 2 (10 SNPs)** | 70.0 | 30.0 |
| **Chromosome 3 (9 SNPs)** | 22.2 | 77.8 |
| **Chromosome 4 (9 SNPs)** | 77.8 | 22.2 |
| **Chromosome 5 (9 SNPs)** | 100.0 | 0.0 |
| **Chromosome 6 (9 SNPs)** | 88.9 | 11.1 |
| **Chromosome 7 (7 SNPs)** | 57.1 | 42.9 |
| **Chromosome 8 (7 SNPs)** | 57.1 | 42.9 |
| **Chromosome 9 (8 SNPs)** | 87.5 | 12.5 |
| **Chromosome 10 (8 SNPs)** | 87.5 | 12.5 |
| **Chromosome 11 (7 SNPs)** | 57.1 | 42.9 |
| **Chromosome 12 (8 SNPs)** | 37.5 | 62.5 |
| **Chromosome 13 (7 SNPs)** | 28.6 | 71.4 |
| **Chromosome 14 (7 SNPs)** | 42.9 | 57.1 |
| **Chromosome 15 (5 SNPs)** | 60.0 | 40.0 |
| **Chromosome 16 (6 SNPs)** | 100.0 | 0.0 |
| **Chromosome 17 (6 SNPs)** | 50.0 | 50.0 |
| **Chromosome 18 (7 SNPs)** | 28.6 | 71.4 |
| **Chromosome 19 (5 SNPs)** | 100.0 | 0.0 |
| **Chromosome X (12 SNPs)** | 58.3 | 41.7 |
| **Total (%)** | **61.1** | **38.9** |

Analysis was performed on DNA isolated from six C57BL/6J;129S1/SvImJ strain mice; SNPs complete background analysis was identical throughout the different mice.

**Supplementary Table 3. Activated biological networks in mice fed a high fructose/sucrose, high fat Western Diet (WD SW) for 8 weeks.**

| No | Network name | Processes | Size | Target | Pathways | p-Value | zScore | gScore |
|---|---|---|---|---|---|---|---|---|
| **1** | Beta-catenin, JAK2, SMAD3, TGF-beta receptor type II, ILK | regulation of cell proliferation (78.4%), positive regulation of cell proliferation (64.7%), regulation of phosphorylation (66.7%), response to organic substance (84.3%), positive regulation of macromolecule metabolic process (78.4%) | 51 | 19 | 21 | 9.82e-14 | 11.73 | 37.98 |
| **2** | MyD88, CaMK IV, IRAK1/2, p38alpha (MAPK14), TRAF6 | energy coupled proton transport, down electrochemical gradient (17.8%), ATP synthesis coupled proton transport (17.8%), mitochondrial ATP synthesis coupled proton transport (15.6%), positive regulation of protein metabolic process (46.7%), phosphorus metabolic process (62.2%) | 50 | 25 | 9 | 4.91e-22 | 16.48 | 27.73 |
| **3** | SURF1, CLCN3, eIF3S7, | hydrogen ion transmembrane transport (23.4%), | 50 | 36 | 0 | 1.74e-41 | 25.27 | 25.27 |

| | | |
|---|---|---|
| C6orf62, RPL9 | proton transport (23.4%), hydrogen transport (23.4%), monovalent inorganic cation transport (23.4%), positive regulation of plasma membrane long-chain fatty acid transport (6.4%) | |

JAK2, Janus kinase 2; SMAD3, SMAD family member 3; TGF, Transforming growth factor; ILK, integrin-linked kinase ; MyD88, Myeloid differentiation primary response gene 88; CaMK IV, Calmodulin-dependent protein kinase IV; IRAK 1/2, IL-1 receptor-associated kinase 1/2; MAPK14, Mitogen-activated protein kinase 14; TRAF6, TNF (Tumor necrosis tumor)-receptor-associated factor 6; SURF1, Surfeit 1; CLCN3, Chloride channel, voltage sensitive 3; eIF3S7, Eukaryotic translation initiation factor 3 subunit 7;C6orf62, chromosome 6 open reading frame 62; RPL9, Ribosomal protein L9.

**Supplementary Table 4. Tumor rate and characteristics.**

| Diet (52 wks) | Predominant type of Steatosis | Degree of Disease | Number of Tumors/Liver | Tumor Rate (%) | Tumor Type |
|---|---|---|---|---|---|
| **CD NW** | None | Normal | None | 0% (0/15) | None |
| **WD SW** | Macrovesicular | NASH | ≥ 3 | 89% (8/9) | .Well-differentiated HCC (8/8)<br>. Poorly-differentiated HCC (3/8)<br>. Adenoma (2/8) |

B6/129 mice were fed for 52 weeks either a chow diet (CD NW), a high fructose/glucose diet (CD SW), a high fat Western diet (WD NW) or a high fructose/glucose, high fat Western diet (WD SW). Values are mean ± SEM for 8-15 mice per group.

**Supplementary Table 5. Comparison of C57BL/6J (B6), 129 S1/SvImJ (S129) and the mixed B6/129 mouse strain at an early stage of steatohepatitis (16-22 wks)**

| | **B6** | | **S129** | | **B6/129** | |
|---|---|---|---|---|---|---|
| **16-22 weeks of diet** | **CD NW** | **WD SW** | **CD NW** | **WD SW** | **CD NW** | **WD SW** |
| **Weight** | | | | | | |
| **- Body weight (g)** | **32.3** ± 0.4 | **43.2** ± 1.6*** | **32.2** ± 1.4 | **36.1** ± 1.4 | **30.8** ± 1.0 | **48.5** ± 1.4*** |
| **- Liver weight (g)** | **1.4** ± 0.1 | **2.9** ± 0.2*** | **1.0** ± 0.1 | **1.6** ± 0.1** | **1.3** ± 0.1 | **3.4** ± 0.2*** |
| **Biochemical parameters** | | | | | | |
| **- AST (U/L)** | **122.0** ± 9.7 | **281.8** ± 64.0 | **42.7** ± 8.0 | **95.8** ± 6.3*** | **73.0** ± 17.3 | **165.0** ± 54.2 |
| **- ALT (U/L)** | **118.7** ± 21.2 | **309.3** ± 64.1 | **35.2** ± 10.1 | **77.5** ± 13.4 | **68.8** ± 16.2 | **151.2** ± 31.2* |
| **- Alkaline Phosphatase (U/L)** | **56.7** ± 4.3 | **105.8** ± 20.5* | **12.2** ± 0.8 | **14.1** ± 1.1 | **10.2** ± 0.2 | **45.2** ± 5.5*** |
| **- Cholesterol (mg/dL)** | **84.7** ± 9.4 | **241.8** ± 17.3*** | **43.0** ± 0.7 | **60.5** ± 2.9** | **19.2** ± 3.9 | **93.7** ± 6.1*** |
| **- LDL-c (mg/dL)** | **33.3** ± 3.4 | **140.9** ± 13.0*** | **9.5** ± 0.6 | **19.5** ± 1.5** | **10.6** ± 2.0 | **60.0** ± 3.0*** |
| **- Triglycerides (mg/dL)** | **64.7** ± 13.0 | **52.4** ± 7.76 | **28.2** ± 3.8 | **26.1** ± 1.5 | **11.6** ± 1.6 | **23.0** ± 5.2 |

C57BL/6J (B6), 129 S1/SvImJ (S129) or B6/129 mice were fed for 16-22 weeks either a chow diet (CD NW) or a high fructose/sucrose, high fat Western diet (WD SW). Values are mean ± SEM for 4-6 mice per CD NW group and 7-13 mice per WD SW group; ***$P < 0.001$, **$P < 0.01$, *$P < 0.05$ WD SW compared to CD NW. ALT, alanine aminotransferase; AST, aspartate aminotransferase; LDL-c, low-density lipoprotein-cholesterol.

**Supplementary Table 6. Activated biological networks in B6/129 mice fed a high fructose/sucrose, high fat Western Diet (WD SW) for 52 weeks as compared to C57BL/6J (B6) mice fed WD SW for 52 weeks.**

| No | Network name | Processes | Size | Target | Pathways | p-Value | zScore | gScore |
|---|---|---|---|---|---|---|---|---|
| 1 | Collagen IV, RPS6, Cyclin D1, Osteopontin, FAK1 | response to growth factor (68.1%), cellular response to growth factor stimulus (66.0%), positive regulation of protein metabolic process (78.7%), cell junction organization (48.9%), positive regulation of cellular protein metabolic process (76.6%) | 52 | 4 | 101 | 8.36e-05 | 7.92 | 134.17 |
| 2 | Activin A, Calsyntenin-3, MMP-2, UGT2B10, Syntenin 2 | transferrin transport (30.6%), ferric iron transport (30.6%), trivalent inorganic cation transport (30.6%), ATP hydrolysis coupled proton transport (28.6%), energy coupled proton transmembrane transport, against electrochemical gradient (28.6%) | 52 | 8 | 24 | 6.83e-11 | 16.49 | 46.49 |
| 3 | CES1, UGT1A9, COL6A1, DCOR, Plastin | flavonoid metabolic process (28.0%), small molecule metabolic process (80.0%), flavonoid process (80.0%), flavonoid glucuronidation (24.0%), flavonoid biosynthetic process (24.0%), monocarboxylic acid metabolic process (48.0%) | 50 | 18 | 0 | 2.34e-33 | 44.75 | 44.75 |

RPS6, ribosomal protein 6; FAK1, focal adhesion kinase 1; MMP-2, matrix metalloproteinase-2; UGT2B10, UDP glucuronosyltransferase 2 family, polypeptide B10; CES1, carboxylesterase 1; UGT1A9, UDP glucuronosyltransferase 1 family, polypeptide A9; COL6A1, collagen, type VI, alpha 1; DCOR, Ornithine decarboxylase

**Supplementary references**

[1] Ayala JE, Samuel VT, Morton GJ, Obici S, Croniger CM, Shulman GI, et al. Standard operating procedures for describing and performing metabolic tests of glucose homeostasis in mice. Disease models & mechanisms 2010;3:525-534.

[2] Contos MJ, Sanyal AJ. The clinicopathologic spectrum and management of nonalcoholic fatty liver disease. Advances in anatomic pathology 2002;9:37-51.

[3] Ludwig J, Viggiano TR, McGill DB, Oh BJ. Nonalcoholic steatohepatitis: Mayo Clinic experiences with a hitherto unnamed disease. Mayo Clinic proceedings 1980;55:434-438.

[4] Bedossa P, Consortium FP. Utility and appropriateness of the fatty liver inhibition of progression (FLIP) algorithm and steatosis, activity, and fibrosis (SAF) score in the evaluation of biopsies of nonalcoholic fatty liver disease. Hepatology 2014;60:565-575.

[5] Kleiner DE, Brunt EM, Van Natta M, Behling C, Contos MJ, Cummings OW, et al. Design and validation of a histological scoring system for nonalcoholic fatty liver disease. Hepatology 2005;41:1313-1321.

[6] Goodman ZD, Becker RL, Jr., Pockros PJ, Afdhal NH. Progression of fibrosis in advanced chronic hepatitis C: evaluation by morphometric image analysis. Hepatology 2007;45:886-894.

[7] Hafkenscheid JC, Dijt CC. Determination of serum aminotransferases: activation by pyridoxal-5'-phosphate in relation to substrate concentration. Clinical chemistry 1979;25:55-59.

[8] Fossati P, Prencipe L. Serum triglycerides determined colorimetrically with an enzyme that produces hydrogen peroxide. Clinical chemistry 1982;28:2077-2080.

[9] Pesce MA, Bodourian SH. Enzymatic rate method for measuring cholesterol in serum. Clinical chemistry 1976;22:2042-2045.

[10] Charuruks N, Milintagas A. Evaluation of calculated low-density lipoprotein against a direct assay. Journal of the Medical Association of Thailand = Chotmaihet thangphaet 2005;88 Suppl 4:S274-279.

[11] Cheung O, Puri P, Eicken C, Contos MJ, Mirshahi F, Maher JW, et al. Nonalcoholic steatohepatitis is associated with altered hepatic MicroRNA expression. Hepatology 2008;48:1810-1820.

[12] Akazawa Y, Cazanave S, Mott JL, Elmi N, Bronk SF, Kohno S, et al. Palmitoleate attenuates palmitate-induced Bim and PUMA up-regulation and hepatocyte lipoapoptosis. Journal of hepatology 2010;52:586-593.

[13] Dillies MA, Rau A, Aubert J, Hennequet-Antier C, Jeanmougin M, Servant N, et al. A comprehensive evaluation of normalization methods for Illumina high-throughput RNA sequencing data analysis. Briefings in bioinformatics 2013;14:671-683.

[14] Ekins S, Bugrim A, Brovold L, Kirillov E, Nikolsky Y, Rakhmatulin E, et al. Algorithms for network analysis in systems-ADME/Tox using the MetaCore and MetaDrug platforms. Xenobiotica; the fate of foreign compounds in biological systems 2006;36:877-901.

[15] Liberzon A, Subramanian A, Pinchback R, Thorvaldsdottir H, Tamayo P, Mesirov JP. Molecular signatures database (MSigDB) 3.0. Bioinformatics 2011;27:1739-1740.

[16] Hoshida Y, Nijman SM, Kobayashi M, Chan JA, Brunet JP, Chiang DY, et al. Integrative transcriptome analysis reveals common molecular subclasses of human hepatocellular carcinoma. Cancer research 2009;69:7385-7392.

[17] Hoshida Y, Villanueva A, Kobayashi M, Peix J, Chiang DY, Camargo A, et al. Gene expression in fixed tissues and outcome in hepatocellular carcinoma. The New England journal of medicine 2008;359:1995-2004.

[18] Hoshida Y, Villanueva A, Sangiovanni A, Sole M, Hur C, Andersson KL, et al. Prognostic gene expression signature for patients with hepatitis C-related early-stage cirrhosis. Gastroenterology 2013;144:1024-1030.

[19] Ahrens M, Ammerpohl O, von Schonfels W, Kolarova J, Bens S, Itzel T, et al. DNA methylation analysis in nonalcoholic fatty liver disease suggests distinct disease-specific and remodeling signatures after bariatric surgery. Cell metabolism 2013;18:296-302.

[20] Hoshida Y, Brunet JP, Tamayo P, Golub TR, Mesirov JP. Subclass mapping: identifying common subtypes in independent disease data sets. PloS one 2007;2:e1195.

[21] Adams LA, Lymp JF, St Sauver J, Sanderson SO, Lindor KD, Feldstein A, et al. The natural history of nonalcoholic fatty liver disease: a population-based cohort study. Gastroenterology 2005;129:113-121.

[22] Rinella ME, Green RM. The methionine-choline deficient dietary model of steatohepatitis does not exhibit insulin resistance. Journal of hepatology 2004;40:47-51.

[23] Yang S, Lin HZ, Hwang J, Chacko VP, Diehl AM. Hepatic hyperplasia in noncirrhotic fatty livers: is obesity-related hepatic steatosis a premalignant condition? Cancer research 2001;61:5016-5023.

[24] Sato W, Horie Y, Kataoka E, Ohshima S, Dohmen T, Iizuka M, et al. Hepatic gene expression in hepatocyte-specific Pten deficient mice showing steatohepatitis without ethanol challenge. Hepatology research : the official journal of the Japan Society of Hepatology 2006;34:256-265.

[25] Fujii M, Shibazaki Y, Wakamatsu K, Honda Y, Kawauchi Y, Suzuki K, et al. A murine model for non-alcoholic steatohepatitis showing evidence of association between diabetes and hepatocellular carcinoma. Medical molecular morphology 2013;46:141-152.

[26] Ishikawa H, Takaki A, Tsuzaki R, Yasunaka T, Koike K, Shimomura Y, et al. L-carnitine prevents progression of non-alcoholic steatohepatitis in a mouse model with upregulation of mitochondrial pathway. PloS one 2014;9:e100627.

[27] Dowman JK, Hopkins LJ, Reynolds GM, Nikolaou N, Armstrong MJ, Shaw JC, et al. Development of hepatocellular carcinoma in a murine model of nonalcoholic steatohepatitis induced by use of a high-fat/fructose diet and sedentary lifestyle. The American journal of pathology 2014;184:1550-1561.

[28] Lee L, Alloosh M, Saxena R, Van Alstine W, Watkins BA, Klaunig JE, et al. Nutritional model of steatohepatitis and metabolic syndrome in the Ossabaw miniature swine. Hepatology 2009;50:56-67.

[29] Ganz M, Bukong TN, Csak T, Saha B, Park JK, Ambade A, et al. Progression of non-alcoholic steatosis to steatohepatitis and fibrosis parallels cumulative accumulation of danger signals that promote inflammation and liver tumors in a high fat-cholesterol-sugar diet model in mice. Journal of translational medicine 2015;13:193.

[30] Wolf MJ, Adili A, Piotrowitz K, Abdullah Z, Boege Y, Stemmer K, et al. Metabolic activation of intrahepatic CD8+ T cells and NKT cells causes nonalcoholic steatohepatitis and liver cancer via cross-talk with hepatocytes. Cancer cell 2014;26:549-564.
